# Cultural, economic, and settlement shifts over the last 9,000 years at Kakapel Rockshelter, Western Kenya

**DOI:** 10.1371/journal.pone.0328805

**Published:** 2025-08-20

**Authors:** Steven T. Goldstein, Natalie G. Mueller, Emma Finestone, Elizabeth A. Sawchuk, Sara Juengst, Anthony Odera Otwani, Jennifer M. Miller, Michelle C. Langley, Ricardo Fernandes, Axel Steinhof, Victor Iminjili, Christine Chepkorir, Anneke Janzen, Christine Ogola, Emmanuel Ndiema, Michael Petraglia, Nicole Boivin

**Affiliations:** 1 Department of Anthropology, University of Pittsburgh, Pittsburgh, Pennsylvania, United States of America; 2 Department of Anthropology, Washington University in Saint Louis, Saint Louis, Missouri, United States of America; 3 Cleveland Museum of Natural History, Cleveland, Ohio, United States of America; 4 Department of Anthropology, Stony Brook University, Stony Brook, New York, United States of America; 5 Department of Anthropology, University of North Carolina Charlotte, Charlotte, North Carolina, United States of America; 6 Kakapel National Monument, National Museums of Kenya, Amagoro, Kenya; 7 Center for Social Sciences, Southern University of Science and Technology, Shenzhen, Guangdong, China; 8 Department of Archaeology, Max Planck Institute of Geoanthropology, Jena, Germany; 9 Australian Research Centre for Human Evolution, Griffith University, Brisbane, Australia; 10 Archaeology, School of Environment and Science, Griffith University, Brisbane, Australia; 11 Faculty of Archaeology, University of Warsaw, Warsaw, Poland; 12 School of Archaeology, Climate Change and History Research Initiative, Princeton University, Princeton, New Jersey, United States of America; 13 Max Planck Institute for Biogeochemistry, Jena, Germany; 14 Department of Anthropology, University of Tennessee Knoxville, Knoxville, Tennessee, United States of America; 15 Department of Archaeology, National Museums of Kenya, Nairobi, Kenya; 16 Department of Earth Sciences, National Museums of Kenya, Nairobi, Kenya; 17 Human Origins Program, Smithsonian Institution, Washington, Columbia, United States of America; 18 School of Social Science, The University of Queensland, Brisbane, Australia; 19 Griffith Sciences, Griffith University, Brisbane, Australia; Tel Aviv university, ISRAEL

## Abstract

The spread of food production in sub-Saharan Africa involved multi-directional dispersals of domesticated plant and animal species, often associated with major migrations. The Lake Victoria Basin of eastern Africa was likely an important crossroads in this process, hosting interactions between diverse populations with hunter-gatherer, mobile pastoralist, and farming lifeways in the Holocene. Recent discovery of a large assemblage of ancient domesticated plant remains at Kakapel Rockshelter in the Chelelemuk Hills of Busia County, western Kenya have provided new insights into the timing for when different domesticated crops were adopted within this key region. Here, we expand on the archaeological and cultural context for these findings by reporting results of field excavations, regional surveys, radiocarbon dating, and artifact analyses for deposits recovered from Kakapel Rockshelter dating over the last 9,000 years. Multiple occupational episodes with distinct cultural and technological traits are apparent including Early Holocene foragers, Early Iron Age agropastoralists, and multiple Later Iron Age populations. Agropastoralism first appears here by c. 2400 BP, but it is not until the introduction of sorghum and finger millet after c. 1200 BP in association with arrivals of new groups with Nilotic ancestry that we document the shift to a higher density of sites and longer-term settlement in the region.

## Introduction

The transition from hunting-and-gathering to agriculture was one of the most profound changes in human evolutionary history. Archaeological, linguistic, and archaeogenetic evidence are revealing that the origins and spread of food production in Africa were spatially and temporally heterogeneous processes. This is especially true for eastern Africa, which saw dramatic changes in food systems and population structures over the last several thousand years. Current models largely focus on major migrations of food producers, beginning with the expansion of mobile herders spreading southward from the Sahara after c. 7500 years before present (BP) [1,2]). Historically, the origins of agriculture in eastern and southern Africa have been associated with the so-called “Bantu Expansion” of iron-using farmers with West-Central African ancestry entering eastern Africa around 2500 BP [3–6]. However, archaeological data show that both pastoralism and farming spread in protracted and non-uniform ways, producing a mosaic of human genetic, cultural, and economic signatures [7–13]. Subsistence categories are also not mutually exclusive, with many populations relying on livestock, crops, and wild resources in different proportions. Furthermore, ethnoarchaeological work reveals the potential for flexibility in populations moving between foraging, farming, and herding within a single lifetime [14–17]. With so many axes of variation, land-use patterns would have been equally heterogenous. Reconstructing changes in human-environmental dynamics across the Holocene therefore requires better recognition of the timing and intensity of change at the local scale.

One of the key regions of economic and cultural coalescence was the broader Lake Victoria Basin, where there is strong evidence for convergence of local fisher-forager lifeways, mobile pastoralism, and agricultural strategies [12,18,19]. This area is also the first location where the three major African domesticated grains of Sahelian sorghum (*Sorghum bicolor*), West African pearl millet (*Pennesitum glaucum*), and eastern African finger millet (*Eleusine coracana*) appear together in the archaeological record [18,20]. Recent archaeological and archaeogenetic evidence from Kakapel Rockshelter on the northeastern margin of the Lake Victoria Basin shows that demographic shifts co-occur with the diversification of early crop economies [13,18]. Here, we report results of archaeological research at and around Kakapel Rockshelter, Busia County, Kenya to better understand the shifts in human behavior, technology, and culture that contextualize major subsistence shifts in the region.

Kakapel Rockshelter is a shallow shelter with an elaborate multi-period rock art panel on the west side of Kakapel Inselberg, within the granitic hills at the base of Mount Elgon, western Kenya. Focused research at Kakapel Rockshelter demonstrates preservation of a well-stratified sequence with four major occupational phases beginning with pottery-making foragers (Phase I: c. 9,400‒3,900 calibrated years before present (cal. BP)) and extending through the Early Iron Age (Phase II: c. 2300‒1790 cal. BP), Later Iron Age (Phase III: c. 1,200‒300 cal. BP), and into pre-colonial/historic occupations (Phase IV: 300 cal. BP to the present). Archaeological datasets from Kakapel and surveys of the surrounding region provide new insights into the diverse populations and economies that contributed to the development of historic, diversified, subsistence systems in the Lake Victoria Basin including the introduction of diverse domesticated plants and animals [18].

## Background

### Holocene foragers and the “Kansyore”

Insolation-driven increases in rainfall along with Early Holocene warming encouraged many hunter-gatherer societies across northern and eastern Africa to begin focusing on rich and reliable aquatic resources. As the largest and one of the most stable lakes in eastern Africa, Lake Victoria supported pottery-using fisher-forager societies from the Early to Late Holocene. These are often referred to as belonging to the “Kansyore” tradition based on similarities in ceramic vessel styles and decorative motifs, use of quartz tools, and apparent focus on lacustrine resources [21–25]. With recent recognition of greater cultural and economic diversity within the “Kansyore” region, there is increasing doubt that a single label can be usefully applied to groups living across such a broad geographic region and temporal range [26]. We use the term “Kansyore” in reference to specific ceramic types published with that label, while acknowledging both the potential diversity of populations producing it and the possibility that future research may subdivide groups beyond current, limited, definitions.

Hunter-gatherer-fisher societies living around Lake Victoria were descendants of earlier, pre-pottery Later Stone Age populations. The beginning of local pottery production marks a major transition in the region’s archaeological record, and the assemblage from Kakapel reported here is among the earliest well-dated Kansyore assemblages. The exact chronology remains unclear as the earliest date for date for Kansyore pottery is not very accurate: a bone apatite date from Luanda with a > 200 year lab error range (GX-8743) suggests that pottery production may have begun as early as the 10^th^ millennium BP [27]. There are also claims of possible (but undated) Kansyore-like occurrences in southern Sudan that might suggest early connections with pottery-making fisher-foragers in the early Holocene “Green Sahara” [28]. The earliest contexts with Kansyore ceramics at Kakapel Rockshelter are now dated to c. 9,300−9,000 cal. BP (see Table 1) but some degree of mixing is possible considering clear evidence of vertical movement of small finger millet seeds through the sequence [see also Table 1]. Despite remaining questions surrounding the start of ceramic production, the practice was widespread around Lake Victoria and western Uganda by 8,500 cal. BP [30–32].

**Table 1:** Radiocarbon dates for Kakapel Rockshelter by trench/occupational phase. Dates are calibrated using OxCal 4.4 software [29] employing a southern hemisphere (ShCal20) radiocarbon calibration curve (Hogg et al., 2020). See Supplemental Text for excavation details of each context (note contexts do not always proceed in stratigraphic order).

Trench	Phase	Context	LabID	Material type	rcybp	error	Cal_Range	Reference
2	IV	104	OS-167169	*Pisum*	210	15	300−101	Goldstein et al. 2024
2	IV	1	SUERC-86058	Human tooth	222	29	311−100	Wang et al. 2020
2	IV	105	P-26848	NID Charcoal	361	19	491−319	*Reported here*
2	IIIb	5	OxA-38808	NID Nutshell	832	21	684-775	*Reported here*
2	IIIb	5	OxA-38807	NID Nutshell	845	21	690-785	Mueller et al. 2022
2	IIIb	2	Wk-47412	*S. bicolor*	848	16	785−695	Goldstein et al. 2024
2	IIIb	4.1	OxA-38796	*S. bicolor*	864	21	897−723	Goldstein et al. 2024
2	III	4	SUERC-86059	Human tooth	895	28	906−731	Wang et al. 2020
2	I	126	SUERC-99031	*C. schweinfurthii*	6463	20	7324-7402	*Reported here*
2	I	9	OxA-38777	NID Nutshell	8233	28	9030-9398	*Reported here*
3	IIIb	105	OS-167170	*Pisum*	920	20	911−752	Goldstein et al. 2024
3	IIIb	6.1	OxA-40174	*B. taurus (collagen)*	901	18	904−734	Goldstein et al. 2024
3	IIIb	3.1	Wk-48696	NID Nutshell	887	18	732-898	*Reported here*
3	IIIb	2	WK-47410	NID Nutshell	813	15	685-731	Mueller et al. 2022
3	IIIa	103.2	Wk-48699	Fabaceae	962	18	923−794	Mueller et al. 2022
3	IIIa	127	OS-162692^†^	*E. coracana*	965	25	925−793	Goldstein et al. 2024
3	IIIa	119	OS-174285^†^	*E. coracana*	975	15	925−798	Goldstein et al. 2024
3	IIIa	2.1^*^	WK-47411	NID Nutshell	977	18	798-926	*Reported here*
3	IIIa	121.1	OS-174283^†^	*E. coracana*	985	15	930−799	Goldstein et al. 2024
3	IIIa	150.2	OS-174281^†^	*E. coracana*	985	20	953−797	Goldstein et al. 2024
3	IIIa	125	OS-174284^‡^	*E. coracana*	995	20	957−800	Goldstein et al. 2024
3	IIIa	125	OS-174282^†^	*E. coracana*	1020	20	959−914	Goldstein et al. 2024
3	IIIa	107	OS-166571^†^	*E. coracana*	1020	20	959−914	Goldstein et al. 2024
3	IIIa	127	OS-162716^†^	*E. coracana*	1060	35	1058−918	Goldstein et al. 2024
3	IIIa	103	P-26847	NID Charcoal	1090	18	936-1057	*Reported here*
3	IIIa	112.2	P-26849	NID Charcoal	1249	19	1121-1272	*Reported here*
3	II	3	WK-47413	NID Nutshell	1713	15	1693−1545	Goldstein et al. 2024
3	II	113	SUERC-97782	NID Nutshell	1780	25	1604-1730	Goldstein et al. 2024
3	II	113	SUERC-94589	NID Nutshell	2232	25	2152-2333	*Reported here*
3	II	10	OxA-40173	*B. taurus (collagen)*	2235	20	2331−2155	Goldstein et al. 2024
3	II	9	Wk-48695	NID Nutshell	2236	18	2331−2155	Goldstein et al. 2024
3	II	9	SUERC-98421	NID Nutshell	2266	29	2345−2156	Goldstein et al. 2024
3	II	118(9)	OS-168214	*V. Unguiculata*	2280	20	2348−2180	Goldstein et al. 2024
3	I/II	156^*^	P-26846	NID Charcoal	2423	35	2350-2699	*Reported here*
3	I	3	SUERC-86057	Human bone	3584	28	3976−3777	Wang et al. 2020
3	I	126	SUERC-99032	*C. schweinfurthii*	6450	25	7335-7425	*Reported here*
3	I	126	SUERC-99030	*C. schweinfurthii*	6510	24	7329-7478	*Reported here*
3	I	121	P-26850	NID Charcoal	6524	25	7334-7501	*Reported here*
3	I	127	SUERC-90741	*C. schweinfurthii*	6575	25	7560−7427	Goldstein et al. 2024
4	IIIb	4	OxA-38810	NID Nutshell	612	26	549-650	*Reported here*
4	IIIa	2.2	Wk-48697	*S. bicolor*	940	18	913−792	Goldstein et al. 2024
4	I	5.1	Wk-48698	NID Nutshell	6222	19	7013-7246	*Reported here*
4	I	5.1	OxA-38811	NID Nutshell	6453	26	7425−7320	Goldstein et al. 2024
4	I	8	OxA-38809	NID Nutshell	8197	28	9274−9025	Goldstein et al. 2024
								
KPLII	III	3	P-26851	NID Charcoal	1154	19	975-1175	*Reported here*

*mixed/disturbed context

†Acid-only pretreatment applied

‡Pooled sample of six finger millet grains prepared with ABA pretreatment

Most sites yielding Kansyore-style ceramics are near the shores of Lake Victoria or along its tributary rivers, leading to the assumption of a fisher-forager focused economy among these populations. Fewer Kansyore occurrences have so far been found in interior regions away from the lake and rivers, including those in north-central Tanzania [33]. Often these are surface finds or low-densities of sherds within otherwise local hunter-gatherer lithic and subsistence signatures. More significant non-lacustrine occupations with similar ceramics are now recognized in western Uganda, where populations may have adopted farming and pastoralism in later periods [26]. These findings align with human archaeogenetic evidence from multiple Early to Middle Holocene human burials in the region which exhibit ancestral components associated with populations living in both the Congo and the Lake Victoria Basins [34]. Kansyore ceramics fade from the archaeological record of the Lake Victoria region with the shift toward Early Iron Age wares starting around 2500 cal. BP, with last occurrences of Kansyore styles in the area by 1,900 cal. BP at Wadh Lang’o and Mumba Rockshelter near Lake Eyasi [19,35]. It is possible these traditions persisted and continued to develop in western Uganda [26].

By the Early Iron Age, food production had arrived in the region from multiple directions. Mobile herders had begun moving into the Lake Victoria Basin from the east c. 2,000 years ago and their material culture appears to overlie Kansyore levels at Wadh Lang’o and Gogo Falls [4,36]. Early Iron Age patterns also spread from the west after c. 2,400 cal. BP along with western African crops like cowpea [18]. Contact and interaction among these diverse peoples were critical in shaping regional trajectories [26] but these dynamics remain obscure in the region.

There has been significant debate over potential changes-through-time in forager economies prior to food production [26,27,37]. Ephemeral early layers at sites like Siror, also around Lake Victoria, indicate an initial emphasis on fishing with only minor use of ceramics until c. 7,500 cal. BP, when ceramic production increases markedly and there is evidence for the emergence of delayed-return seasonal land-use patterns [31]. This includes evidence for longer occupations at dry-season lake-coasts where people relied on shallow-water fishing and shellfish collecting. [27,32,37,38]. Large shell middens at forager sites are the result of sustained shellfish collection during either repeated occupations or semi-sedimentary use at these locations. People making Kansyore ceramics appear to have used river-rapid sites during the wet seasons to take advantage of fish spawning [16,38]. Likely use of weirs and recovery of bone-points at these sites demonstrate greater investment in specialized fishing strategies [31,36]. Kansyore pottery at rockshelters further from the lakeshore have also prompted speculation for seasonal terrestrial hunting within this economy [22,39]. Their development of a delayed-return economy around Lake Victoria with seasonal scheduled use of lacustrine and riverine resource and long-term coastal habitations may have been a response to the onset of more arid and seasonally variable conditions beginning c. 8,000 cal. BP [40].

Around 5,000 cal. BP settlement strategy appears to shift in riverine areas and few lakeshore coasts from this time have been found. Until recently, it was unclear when or how rapidly this change occurred as few sites from the so-called “Middle Kansyore” phase had been excavated. Recent research at the shell midden site of Namundiri A in Uganda demonstrates continuation of lake-shore occupations where people relied on aquatic foods but supplemented their diets with terrestrial hunting from c. 6,500−6,000 cal. BP [41]. This shift presages a later and more rapid economic adjustment in favor of terrestrial resources coincident with peak aridity at the termination of the African Humid Period through the Mid-Holocene. While this climate shift may not have had a major impact on Victoria lake levels, paleoclimatic reconstructions indicate regional environmental shifts that may have impacted wild resource availability to the degree that foragers were compelled to change their land-use practices [42,43]. Dietary shifts involving a larger component of terrestrial mammal hunting coincide with a proposed shift in Kansyore ceramic styles. Whereas earlier period pottery is characterized by impressions and punctates, zig-zag motifs made by incision and rocker-stamp techniques become more common over time [37]. So far, there has been no indication of lithic technological change through the Holocene, and material continues to resemble typical Later Stone Age toolkits that emphasize backed pieces and small scrapers [44,45].

Over the last ~1,000 years there is evidence of forest-decline around Lake Victoria despite an overall rise in lake levels. This mismatch between paleoclimate and paleoecology data suggests that deforestation and increased run-off was caused by agricultural land-clearance by this time [40,43,46]. Contact with incoming herders by at least 2,000 cal. BP also resulted in Kansyore-producing hunter-gatherers acquiring livestock, although it is unclear if this interaction led to integration of animal management or adoption of pastoralism [12,38]. It was also around 2,500–2,000 cal. BP that forager communities likely began interacting with Bantu-speaking groups who produced iron and small-scale agriculture [12,18,19,47].

### Early herding and the “Pastoral Neolithic”

After 7000 cal. BP, increasing aridity at the termination of the African Humid Period in northern Africa pushed pastoralists south of the Sahara [1,17,48]. The first evidence for people who managed livestock in eastern Africa comes from the Lake Turkana Basin of northern Kenya, c. 5,000 cal. BP [49–51]. In southern Kenya, an early “trickle” of livestock has been detected at hunter-gatherer sites in the Central Rift Valley by 4000 cal. BP [7,52,53]. Two individuals excavated at PretteJohn’s Gully near Lake Nakuru and dated to c. 4,000 cal. BP have shared ancestry with later “Pastoral Neolithic” (PN) herders but were not modelled as directly related. [11] The spread of herding into the Central Rift involved multiple migrations of small groups of herders who may have been at least somewhat genetically and culturally distinct.

During the PN, ~ 3200 cal. BP, sites with large proportions of domesticated livestock remains and indicators of primary reliance on herd management become widespread across the Central Rift and Athi-Kapiti Plains [7]. Signatures of these PN economies then appear across the Loita-Mara of south-western Kenya by 2,700 cal. BP [54,55]. By this time there are clear divergences in material culture and technological patterns that have led to the categorization of “Elmenteitan” and “Savanna Pastoral Neolithic” traditions within the PN, thought to reflect different social or economic group identities despite tight genetic clustering of sampled PN individuals across these divisions [52,56–59].

The earliest arrivals of herding further west towards Lake Victoria are less clear. As suggested above, initial interactions between herders and local foragers could have begun as early as 3,000 cal. BP. Along the northern shore of Lake Victoria, obsidian sourced to the Central Rift Valley increases in frequency at fisher-forager sites after 2,000 cal. BP, suggesting more regular herder-forager interactions by that time [60]. The first detectable pastoralist habitations in the southern Nyanza portion of the lake basin at Gogo Falls and Wadh Lang’o date to c. 1,900–1,700 cal. BP [4,36,61]. At both sites, PN levels overlie occupations with Kansyore pottery, indicative of some level of replacement or displacement associated with the gradual spread of herding lifeways. So far there has been no evidence of a PN presence on the north side of Lake Victoria, but new evidence from Kapsoo Rockshelter suggests that by c. 2,000 cal. BP herders were occupying the nearby Uasin Gishu Plateau [62].

### Early Iron Age (EIA) mixed-farming

The arrival of farming to the Lake Victoria Basin (and eastern Africa generally) is often associated with the beginnings of the Iron Age around 2,500 years ago. Iron tools and new styles of incised-line pottery with beveled rims and basal concavities (called “dimple-based”) enters the African Great Lakes region from the west possibly associated with the “Bantu Expansion, “though this relationship remains contentious [47,63,64]. This distinct new “Urewe” ware and iron smelting may have expanded into Rwanda and Burundi after 2,500 years ago [47,65,66]. However, the basis for Urewe chronology is radiocarbon dated charcoal, often from highly problematic contexts and with large error ranges, making chronological interpretations problematic [22,47].

Associations between EIA pottery and food production rely on few data points. One of the few direct associations between Urewe ware and West African crops comes from Kakapel, where a feature with Urewe pottery and West African cowpea are dated to 2,300 cal. BP at Kakapel Rockshelter [18].Urewe sites in western Rwanda have evidence of sorghum, pearl millet, and cowpea after 1,700 cal. BP [20]. This is roughly consistent with pollen and agricultural fallow evidence for intensified farming after 1,700 cal. BP [67]. Co-occurrence with West African crops supports a connection to Bantu-speaking migrants, but it is not possible to determine how much they relied on domesticated plant foods overall. The widespread adoption of finger millet and its greater density at sites after 1,300 years ago likely indicates a shift toward more intensive farming at least by that point [20,68].

Urewe traditions spread quickly around the southern flanks of Lake Victoria into Tanzania but appear much later along the northern lake Basin into western Kenya. Dates for Urewe horizons around the Winam Gulf indicate the earliest arrival of iron-using farmers occurred from c. 1500–1300 cal. BP [4,22,47,69,70]. Settlement patterns are similar to those of earlier foragers, with sites located along major rivers into the lake or near the lake shore. Urewe levels generally overly those of PN and/or Kansyore occupations [36,70]. The end of the Urewe phase is poorly dated, but usually considered to occur in the later part of the second millennium BP. Some sites in Uganda show a “devolved” or “transitional” Urewe that may indicate increased regionalization or social instability [19,63,71]. This is coincident with a broader paucity of Iron Age sites across the Congo Rainforest and Great Lakes which has been contentiously associated with a regional population collapse [72].

### Later Iron Age (LIA) agro-pastoralism

The beginnings of the Later Iron Age (LIA) phase are associated with a re-organization of subsistence and land-use associated with another wave of Nilotic agro-pastoral communities after c. 1,000 cal. BP [69,73]. This change occurred against the backdrop of growing climatic instability around Lake Victoria [74]. This phase of “Roulette” decorated pottery followed a northeast-to-southwest spread, with earlier manifestations in Iron Age Kenya, possibly reflecting population movement along the Ugandan highlands. Once Roulette traditions reached the Great Lakes Region, they became clearly associated with increasing specialization, political centralization, and the formation of Bantu kingdoms [6,75–77]. As a result, the Roulette phase is thought to also mark an increase in specialized farming and livestock management around the Great Lakes region; however, there have been few focused zooarchaeological studies of Roulette sites, and recovery of archaeobotanical remains has been limited [78]. Recently, recovery of a large assemblage of diverse domesticated and wild plant remains from Kakapel Rockshelter supports work from Rwanda demonstrating the use of sorghum and finger millet by this time [18,20]. The Kakapel dataset shows increasing focus on finger millet through the LIA, as well as use of the domesticated field pea [18,68]. Emerging data therefore indicate the LIA was a period when economies more reliant on grain agriculture developed in the region, but with continued use of domesticated livestock and wild plant and animal resources.

## Materials and methods

### Kakapel Rockshelter, excavations, and surveys

**Fig 1:**
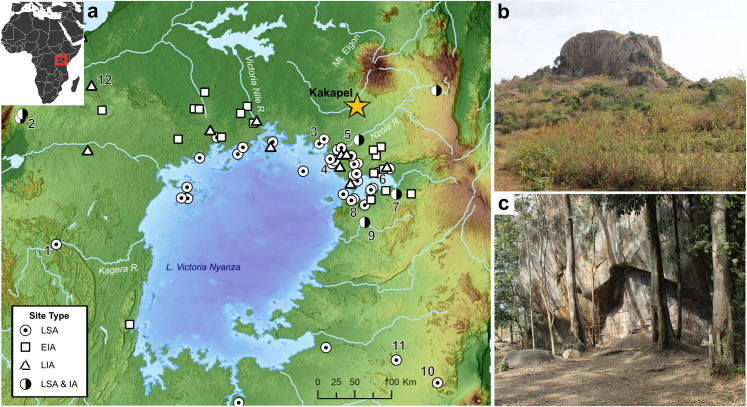
Location and setting of Kakapel Rockshelter; (a) Kakapel Rockshelter relative to other Later Stone Age (LSA), Early Iron Age (EIA) and Later Iron Age (LIA) occurrences around Lake Victoria; (b) Kakapel inselberg; (c) Kakapel Rockshelter overhang and main site area. Select sites labeled in (a): (1) Kansyore Island; (2); Ndali Crater Lakes Region sites; (3): Lugala and Namundiri; (4) Pundo and Usenge; (5) Siror; (6); Rangong; (7); Wadh Lang’o; (8); White Rock Point and Luanda; (9); Gogo Falls; (10) Nasera Rockshelter; (11) Seronera; (12) Munsa. Basemap generated from NASA Shuttle Radar Topography Mission (SRTM) data accessed from USGS Earth Explorer (CC BY 4.0). Africa inset from Wikipedia (CC BY-4.0).

Kakapel (from *Kakapeli* in Teso) is a granitic inselberg within the Chelelemuk Hills in North Teso, Busia County, of western Kenya. The site is located at around 1,420 m asl at the base of Mt. Elgon, just outside the margins of the Lake Victoria basin drainage. This is one of the highest rainfall regions of Kenya, receiving an average of 1,250–2,000 mm of precipitation distributed across two rainy seasons per year. Erosion of the rock surface has created several sheltered areas of various sizes and small pseudo-caves around the entire perimeter of the inselberg. The primary Kakapel Rockshelter I prehistoric site is located along the western side of the inselberg, where there is a panel of preserved Iron Age rock art (**Fig 1**). Archaeological strata are densest under the approximately 5 m deep and 4–10 m tall overhang that forms the shelter but they extend at least an additional 8 meters west outside the shelter. While the rock shelter is not very large, most of it remains dry, even during major rainfall.

The ground in the vicinity of the rock art panel has extensive evidence for prehistoric occupation and is where archaeological excavations have been focused. There are numerous other potential sub-sites within a few hundred meters of the main rockshelter, including several smaller shelters and pseudo-caves formed by massive rockfall. Kakapel is currently a protected site managed by the Trust for African Rock Art (TARA) and the National Museums of Kenya (NMK). It is surrounded by mixed-open forest and small patches of pasture where cattle are occasionally left to graze. Much of the surrounding area is now used for agriculture, with people growing diverse crops including maize, tobacco, amaranth, peanuts, cassava, and finger millet. Wild finger millet (*Eleusine corcana ssp. africana)* is ubiquitous in the area, both in residential areas and agricultural fields.

When first described, rock art was restricted to a single 4 x 2.5 m panel depicting humans, large antelopes and buffalos, a scene of elephant hunting, as well as individual spirals, tortoise shell designs, and circular figures with both red and white phase figures [79]. These were noted to be the best-preserved instances of rock art in the region. Subsequent surveys in the region identified three additional rockshelters/pseudocaves that were “littered with potsherds and grinding stones”, as well as iron smelting sites [80].

**Fig 2:**
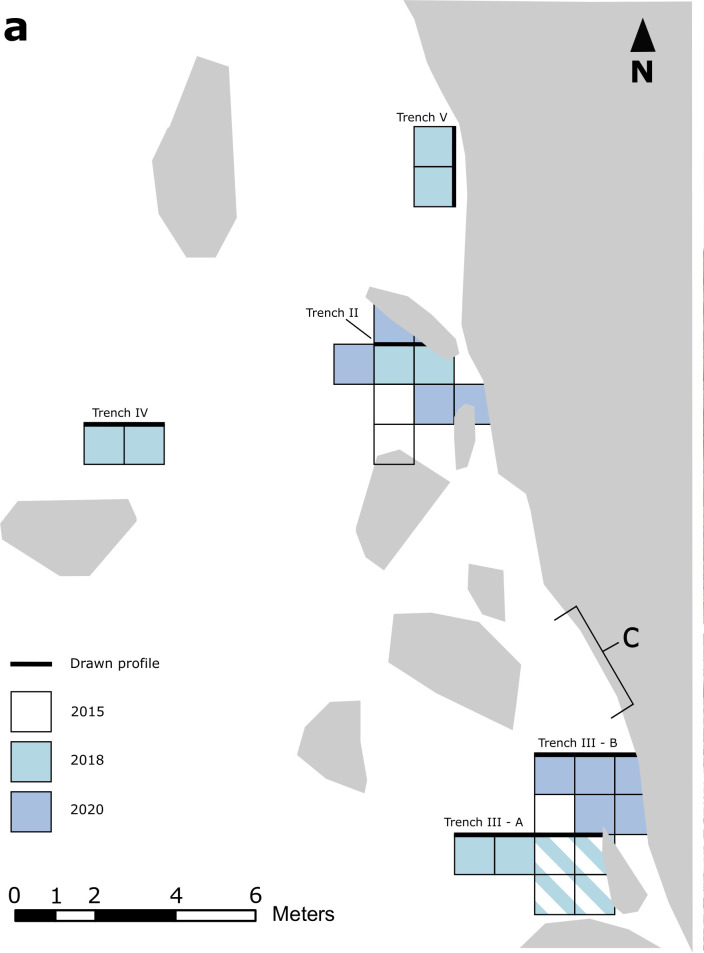
Excavation plan of Kakapel Rockshelter main excavation area.

The first excavations at Kakapel Rockshelter took place in 2012 with a single 1x1 m trench (Trench I). Trench I was placed in the vicinity of the later Trench IV, but the original datum could not be relocated, and so the precise location of Trench I could not be determined. In preparation for the installation of a fence to protect the rock art in 2015, two more trenches (Trenches II and III) were opened roughly in line with the shelter dripline where the fence was to be placed (Fig 2). These excavations revealed a deeper sequence with a greater diversity of pottery and detected *in situ* human remains, which were not excavated at that time.

In 2018, excavations resumed as part of collaborations between the NMK and the Max Planck Institute for the Science of Human History. The 2018 excavations expanded the existing trenches, with the addition of Trench IV further away from the shelter to detect the spatial extent of deposits and Trench V further north along the shelter wall. Fieldwork in 2020 involved further expansion of Trenches II and III (Fig 2) as well as survey and testing of additional rockshelters at Kakapel and nearby inselbergs.

Excavations followed standard approaches for eastern Africa. Excavations proceeded in arbitrary 5–10 cm increments within 1x1 m grid squares unless changes in sediment color or texture, artifact density or styles, or other detectable features were identified. In such cases, natural or anthropogenic boundaries were used to separate horizontal units. Each homogenous layer, discrete natural feature, or discrete archaeological feature was excavated as a separate context and each context was assigned an independent number within the trench. To maximize the potential for archaeobotanical recovery, flotation samples were taken from almost all contexts, with 100% flotation of the matrix from all archaeological features. All samples were processed in the field via the bucket flotation method through a 2-micrometer mesh with heavy fraction recovered from a 2 mm screen.

A LEICA™ total station was used to record the outline and top and bottom elevations for all contexts (including arbitrary spits, archaeological features, and large rockfall) in three-dimensional space. Arbitrary flotation samples that were not part of a discrete context were mapped with only top and bottom elevation points. Piece-plotting began 10 cm below surface and all artifacts 2 cm or larger were also mapped with the total station. All sediment not removed for flotation or geological study was sieved through a 2 mm mesh to recover all cultural and natural material not mapped *in-situ*.

In 2020, a field survey was initiated by E.F., E.E., and A.O. to further characterize the Kakapel inselberg and rockshelters in the Chelelemuk Hills. Survey efforts were directed at locally known rockshelters and their immediate vicinities. We documented the location of nearby rockshelters and shallow caves that preserved evidence of human occupation. The presence of cultural artifacts on the surface such as pottery, bone, lithics, grinding stones, or rock art were recorded at each rockshelter.

### Radiocarbon dating

Chronology for contexts and horizons was established through AMS radiocarbon dating. When possible, short-lived botanical samples (nutshell, seeds, other identifiable plant parts) were chosen for dating, and charcoal samples of unidentified wood species were selected only when other macrobotanicals were not available. Samples of bone were submitted for dating only when direct dates for human remains or domesticated animal remains were necessary. Unless otherwise noted, sample pretreatment, combustion, and reduction protocols followed published standards for the Oxford Radiocarbon Accelerator Unit, UK (OxA) [81], Waikato Radiocarbon Dating Laboratory, New Zealand (Wk) [82], SUERC Radiocarbon Lab, Glasgow, Scotland (SUERC) [83], National Ocean Sciences Accelerator Mass Spectrometer, Woods Hole, USA (OS) [84,85], and Max Planck Institute of Biogeochemistry (P) [83]. Several individual grains of finger millet (Eleusine coracana) were deemed too small to yield enough carbon following a standard acid-base-acid (ABA) treatment and were subjected to an acid-only pretreatment and these are noted in Table 1 (see also [18]). Radiocarbon dates were calibrated using OxCal 4.4 software [29] employing a southern hemisphere (ShCal20) radiocarbon calibration curve [86,87].

## Results

### Kakapel occupational sequence and chronology

The surface of Kakpel Rockshelter follows the natural eroding regolith in sloping roughly 30 degrees south-to-north, and roughly 10 degrees east-to-west across the extent of excavated areas. This difference is most pronounced in Trench III, and least evident in Trenches IV and V. In combination with large rockfall from the shelter, and deeper depositional pockets forming between the rockfall, the surface topography of the site at any period in its history would have been uneven. Excavations at Kakapel also identified a high number of natural and anthropogenic features, including pits that cut into underlying contexts. As a result of all these circumstances, it is not possible to present a single stratigraphic sequence for the entire site. Sequences captured in Trenches II-V are considered separately to understand the complex depositional history of the site. A summary of the radiocarbon dates used to assemble chronologies for each trench are presented in Table 1 (see also Goldstein et al., 2024).

#### Trench II.

**Fig 3:**
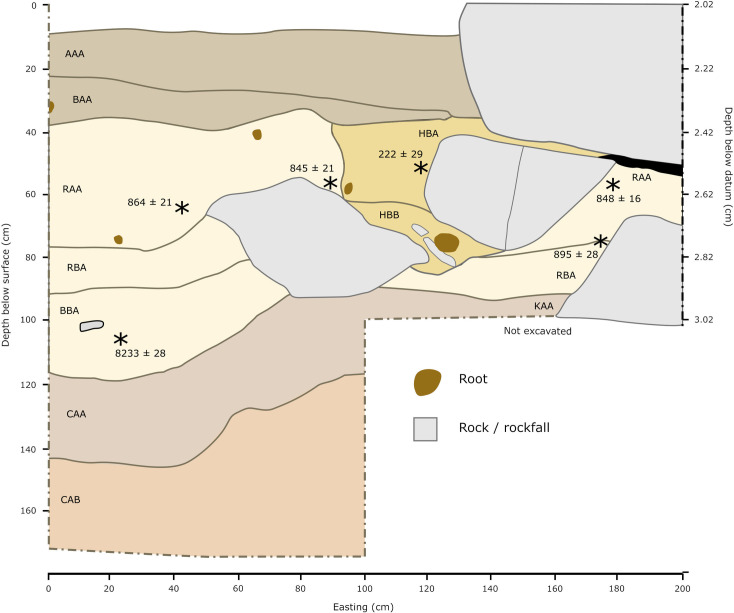
Stratigraphic profile of Trench II. Stars mark approximate depths and locations of radiocarbon date samples. See Supplemental Text for stratigraphic level descriptions.

**Fig 4:**
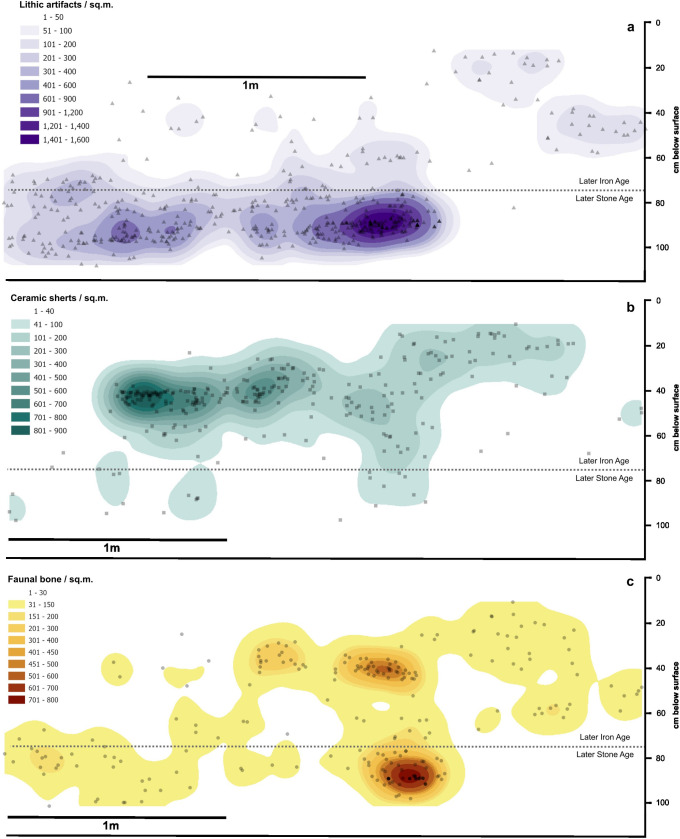
Horizontal distribution and kernal density visualizations of lithics, ceramics, and faunal bone in Trench II (viewing the easting profile).

Located near the center of the site, the upper portions of Trench II contain LIA deposits featuring a high density of Roulette pottery along the edge of the large rockfall that marks the trenches northern border (Figs 3 and 4). The upper ~20 cm of the trench contain mixed historic pottery and near the bottom of this horizon is the start of a burial pit uncovered in 2016. A radiocarbon date on the individual of c. 200 BP indicates that material above the burial reflects LIA-to-historic use of the shelter. Below this burial feature level, artifact density is highest in the easternmost meter of the trench with a more continuous distribution of pottery, lithics, and bone until dense rockfall is encountered. Several small burnt patches and possible hearths were detected in this area that yielded a large assemblage of domesticated grains (see below). These macrobotanical remains and an isolated human tooth from these contexts produced several dates indicating rapid deposition during an earlier phase c. 685–800 cal. BP (see Table 1).

Moving west and out of the sheltered area, the boundary between a ~ 25 cm thick horizon dominated by historic pottery and a lower horizon with small quantities of Kansyore pottery and more abundant lithics and bone becomes clearly defined within the stratigraphy (Fig 4). Artifact distributions across the entire trench provide a clearer picture of an apparently stable surface sloping gradually from the shelter westward. Approximately 15 cm below these contexts was a second surface marked with burnt features and a likely hearth containing nutshell fragments dating to 9,027–9,286 cal. BP (OxA-38777, 8233 ± 28 rcybp). One sherd with likely Kansyore decoration and several undecorated pot sherds were found at this depth, consistent with the earliest dates for Kansyore material around Lake Victoria. Quartz lithic artifacts declined over the next 40 cm until reaching sterile compacted eroding regolith.

#### Trench III.

**Fig 5:**
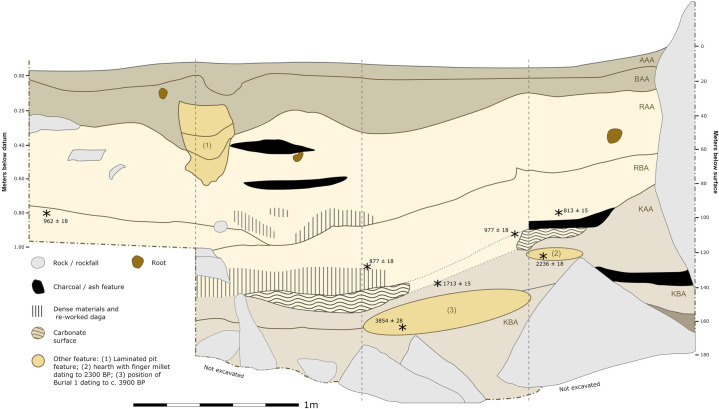
Northern profile of Trench III 2016-2018 excavations (profile III-A). Modified from Goldstein et al., 2024. Contexts with Roulette pottery are prefixed with R-, contexts Kansyore pottery with K- (see Supplemental Text for complete stratigraphic descriptions).

**Fig 6:**
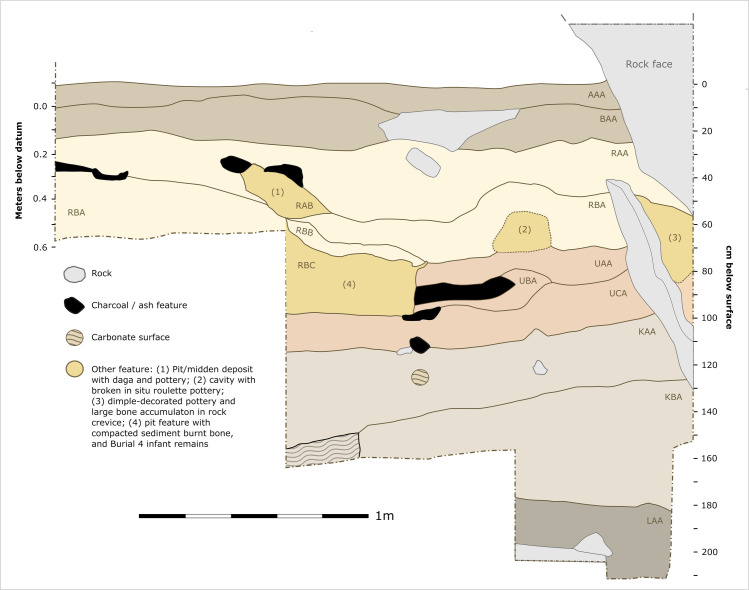
Northern profile of Trench III 2020 excavation extension into the back of the rockshelter (profile III-B). Contexts with Roulette pottery are prefixed with R-, contexts with Urewe pottery with U-, and Kansyore pottery with K- (see Supplemental Text for complete stratigraphic descriptions). Modified from Goldstein et al., 2024.

Trench III is located at an elevated area under the most protected portion of the current rockshelter. It also has the most complex archaeological sequence and the greatest density of features, burials, animal bone, and pottery. Both the 2018 and 2020 profiles are presented (Figs 5–6) as they capture different occupational components.

Like Trench II, the upper 20 cm of this trench features mostly mixed historical debris including ethnohistoric pottery and glass, as well as several large root disturbances. Below this are LIA layers containing Roulette pottery with multiple radiocarbon dates reflecting formation between 680–920 cal. BP (Table 1). Rapid accumulation of the LIA components here is likely related to more intensive (but not necessarily long term) occupations as evidenced by the high number of hearth features, ash dumps, pits, and dense artifact concentrations (Figs 5–6). Burial 3 was encountered within this stratum and appears to date to the LIA.

The lower Roulette stratum (RBA in Fig 6) is marked by a clear horizon of denser pottery particularly in the back of the shelter. These contexts yielded all examples of LIA pottery (see below). This collection includes a single large vessel broken *in-situ* under a large tabular rock. At the start of this stratum, a narrow crevice formed by rock spall that had incompletely detached became visible. It appears this crevice was used as a midden deposit contemporaneously with the LIA (Fig 6, feature ‘3’, and fragments of a large, spouted, open bowl, a large bovid mandible, and several other large animal bone fragments were found within it. Finally, a large flat-bottomed pit feature was bisected along the northern half of the unit that extended from this horizon into underlying contexts (Fig 6, feature ‘4’). This area was noticeably more compacted than the surrounding matrix and was rich in charcoal and burnt bone fragments. Most of the bone in the pit was faunal but also included several burnt fragments of a partial human cranium and mixed post-crania. It is not clear if the partially cremated infant remains were mixed into this deposit intentionally or were disturbed and redeposited into the pit later.

Contexts below the LIA strata were not continuous horizons. Excavations encountered significant variation between areas closer to the shelter wall, areas under the shelter overhang but away from the wall, and areas outside the shelter. In areas within 1.5 m of the shelter wall, excavations uncovered a rectangular compacted feature 1.5 m long and 0.5 m wide, however; it was partially bisected by the LIA pit containing burial 3 (discussed below). Based on two small charcoal features resting on top of the rectangular feature and the density of pottery surrounding it, it is likely related to human habitation. This context and the area immediately surrounding it were the only parts of the site where numerous fragments of diagnostic Urewe pottery were recovered near each other and so it is attributed to a minor EIA occupation at Kakapel. Urewe-bearing deposits were not continuous but clearly thinned and then disappeared moving away from the shelter wall such that they were not detected in Profile III-A (Fig 5).

In areas further south and west from the back wall of the shelter there was a calcium-carbonate surface at the base of LIA contexts and immediately overlying Kansyore-bearing contexts (as supported by radiocarbon dates). The carbonate context dips sharply east-to-west and gradually south-to-north and so interfaces other strata at different points across the trench (S1 Fig). At its southern extent, the upper surface is smooth with embedded charcoal fragments and several ash and hearth features directly on top of it, which together indicate this was a living surface during the LIA. As the carbonate feature was traced north and further down, it became more uneven with a rougher surface that exhibited what resembled desiccation cracks (S2 Fig). Additional burnt features were encountered across the entire surface. Based on the position of the carbonate feature directly under the drip-line of the shelter, we hypothesize that the surface is a natural product of concentrated water percolation and carbonate aggregation that formed after the deposition of EIA materials in the shelter.

**Fig 7:**
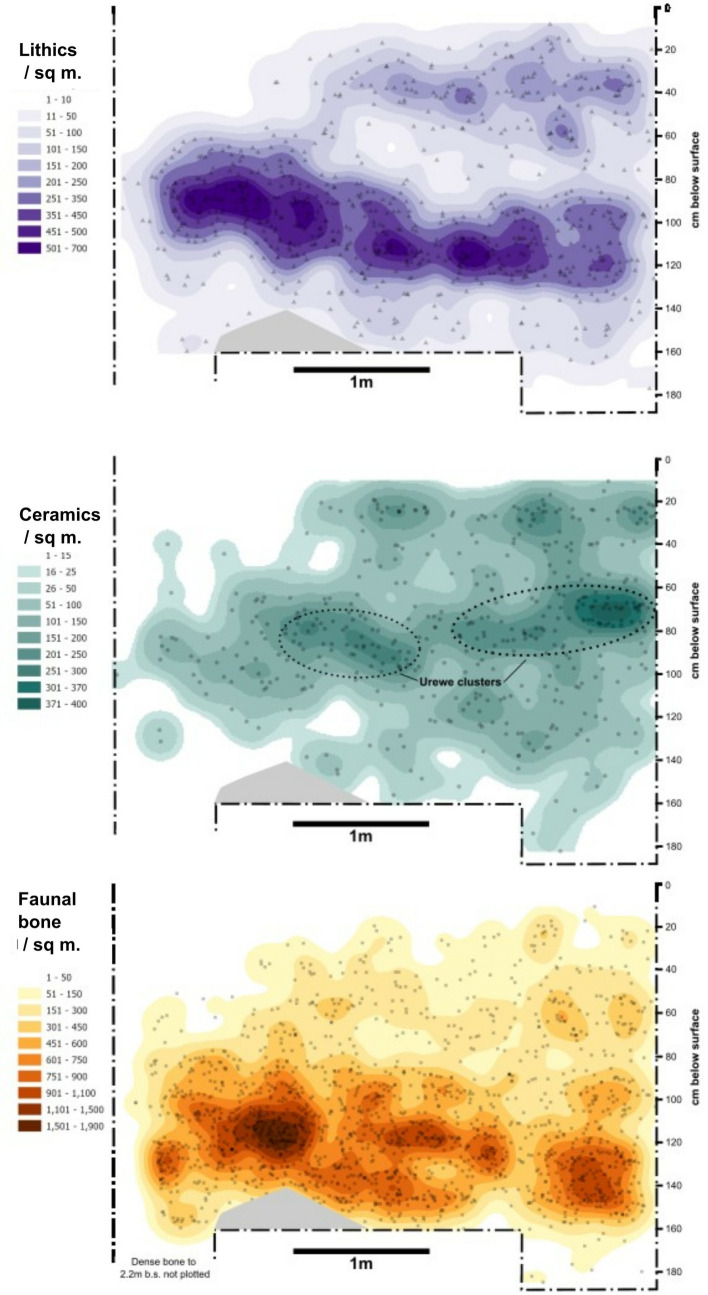
Horizontal distribution of plotted locations and kernel density visualizations of lithic, ceramic, and bone artifacts in Trench III viewed along the easting.

Burnt features under the carbonate surface dated to before 2,500 cal. BP and represent the start of unambiguously Holocene LSA/forager contexts (layers KAA and KBA in Figs 5–6). Most of the deposits below this point dated to between 7,000–7,500 cal. BP (Table 1) and contained much higher densities of bone and lithics while containing lower frequencies of pottery (Fig 7). A human burial (Burial 1) within this deposit dated to 3725–3972 cal. BP with signs of a clear burial pit indicates later intrusion (see below). Quartz lithics and Kansyore pottery sherds were found in the matrix of the pit, but not in direct association with the human remains.

At 150 cm below surface, materials began to diminish significantly across most of the trench and likely taphonomic fauna including small rodent bones and articulated snake vertebrae become more common as denser rockfall blocked further excavation. A gap in the rockfall layer in the northeast corner of the trench allowed continued excavation of a 50 cm^2^ unit to a depth of 210 cm below surface. This context (Fig 6: Layer LAA) contained only dense deposits of primarily mammal bones (faunal material was so dense it was not plotted).

#### Trench IV.

**Fig 8:**
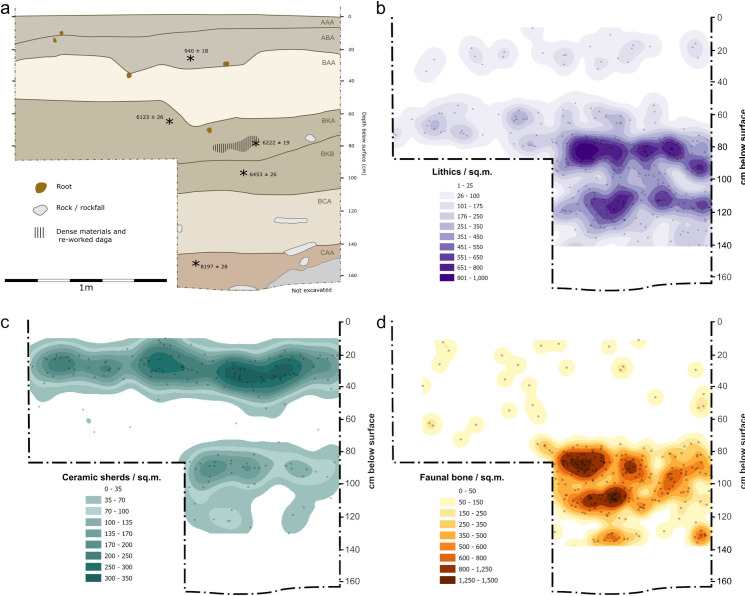
Horizontal distribution of plotted locations and kernel density visualizations of lithic, ceramic, and bone artifacts in Trench IV viewed along the northing. Note the separation between upper Later Iron Age strata with more frequent ceramics and lower Kansyore zone with more abundant lithics and fauna.

Trench IV was excavated further west of the main rockshelter at the edge of the largest visible rockfall to determine if earlier deposits could be detected further from the current shelter-line. This trench featured the most dramatic division between the Later Iron Age levels dominated by Roulette pottery within the top 30 cm (680–730 cal. BP) and the dense LSA levels that began at ~70 cm below surface (Fig 8). There is a clear gap between Iron Age and LSA horizons at this part of the site that is comparable to the pattern in the westernmost portions of Trench II, but not detectable in Trench III. Near the top of the LSA artifact horizon was a noticeable surface with lithics, numerous hammer stones and pitted-anvils, as well as amorphous accumulations of fire-hardened clay. Dates from the top, middle, and bottom of this dense layer indicate formation between 6855–7425 cal. BP. Lithics and diminishing proportions of animal bone continued to be present until excavations reached dense rockfall on top of eroding regolith material, but macrobotanical material at this layer indicated human presence by 9025–9273 cal. BP, like the basal dates for Trench II.

#### Trench V.

Trench V was the northernmost trench of the main site in line with the narrowing dripline of the shelter. The first 40 cm were dominated by a large ash feature overlain by a blackened charcoal-rich feature associated with large fragments of animal bones identified as *Bos taurus*. Several bones exhibited signs of burning. Roulette and historic pottery were mixed across this horizon, indicating formation within the last few hundred years. As with Trench II and IV, there was then a gap of ~30–40 cm before encountering a dense layer of lithics debris, but no pottery. After a depth of 70 cm below surface, the sediment transitioned to an indurated clay with very poor bone preservation, likely reflecting movement of the water table. This area is also the lowest point of the site, and we believe water draining from the remainder of the site collects at these levels. Sediment became so compacted that excavations were terminated at 1 m below surface.

#### Kakapel Rockshelter II test pit.

In 2020, another small shelter along the north-western corner of the inselberg was tested. This area is only roughly 3 x 6 m, formed by a large spall of rockfall (S3 Fig a). The 1 x 1 m trench extended 75 cm below surface before encountering eroding bedrock. The stratigraphy was irregular with many undulations and non-continuous contexts (S3 Fig b). Some disturbances were attributed to animal burrowing, however; there was also a large charcoal and ash-rich feature near the bottom of the trench that indicated human activity also played a major role. While neither stratigraphy nor artifact distributions indicated any clear horizons or occupational phases, lithics, animal bone, and Iron Age type pottery were found throughout the sequence, and a radiocarbon date on charcoal from the lower feature returned a date of 975–1175 cal. BP (P-26851, 895 ± 28 rcybp) (Table 1). One possible Kansyore sherd was present at the very bottom of the trench but there were no other indicators of pre-Iron Age use of this area.

### Surveys

**Fig 9:**
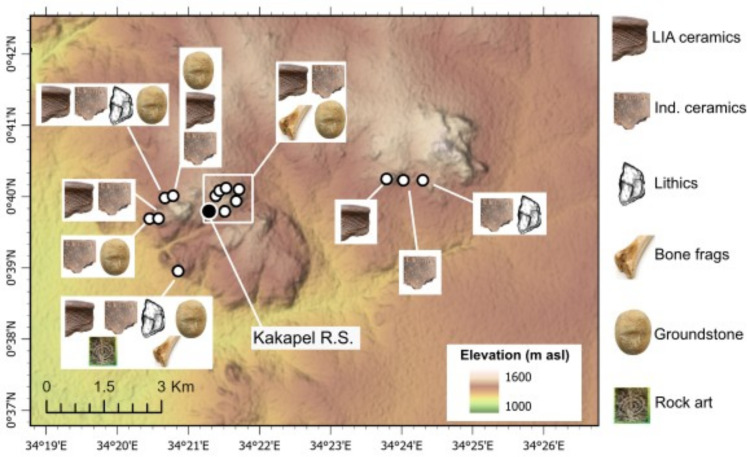
Locations of the 14 rockshelters identified through survey in the Kakapel area (see Fig 1). At each locality the presence of cultural artifacts (roulette pottery, indt. [indeterminate] pottery, bone, lithic, grinding stone, and rock art) is shown. Basemap generated from NASA Shuttle Radar Topography Mission (SRTM) data accessed from USGS Earth Explorer (CC BY 4.0).

Fourteen rockshelters with evidence of occupation were identified around the Chelelemuk Hills. These rockshelters are all within a 5 km radius of Kakapel, and six are located within 300 m of the Kakapel excavations (Fig 9) suggesting that a variety of rockshelters may have been occupied in this region in the LIA and Historical Phase (Table 2).

**Table 2:** The latitude and longitude (in degrees, minutes, seconds), and elevation (meters above sea level) of the 14 rock shelters surveyed in this study. Surface finds of lithics, ceramics, bone, and grinding stones and/or presence of rock art is noted for each locality.

Location			Material culture present				
*Latitude*	*Longitude*	*Elevation (m.a.s.l.)*	*Lithic*	*Ceramic*	*Bone*	*Grinding Stone*	*Rock Art*
0°40’6.96“N	34°21’30.96“E	1431		Y			
0°40’6.96“N	34°21’29.16“E	1433		Y	Y		
0°40’6.49“N	34°21’35.09“E	1412	Y	Y	Y	Y	
0°40’5.28“N	34°21’33.06“E	1424		Y			
0°40’5.16“N	34°21’29.16“E	1439		Y		Y	
0°40’2.04“N	34°21’29.93“E	1472		Y		Y	
0°40’15.82“N	34°23’40.87“E	1416		Y			
0°40’14.87“N	34°24’8.28“E	1376	Y	Y			
0°40’14.19“N	34°23’52.29“E	1460		Y			
0°39’57.74’‘N	34°20’47.93’‘E	1439		Y		Y	
0°39’56.76’‘N	34°20’46.33’‘E	1423		Y	Y	Y	
0°39’37.51“N	34°20’39.15“E	1390		Y			
0°39’36.87“N	34°20’35.65“E	1362		Y		Y	
0°38’58.81“N	34°20’53.02“E	1348	Y	Y	Y	Y	Y

**Fig 10:**
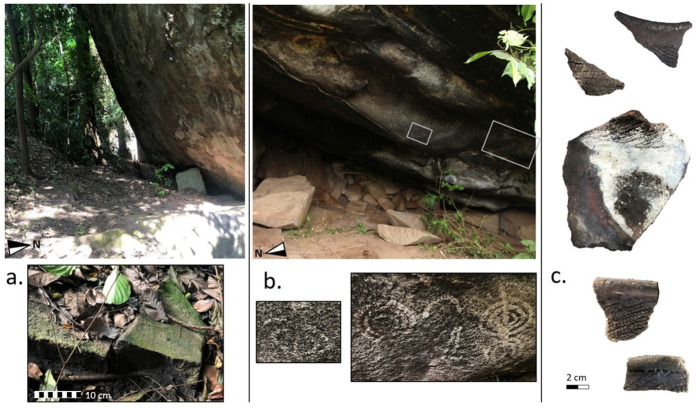
Rockshelter sites identified during surveys; (a) one of the rockshelters in the immediate proximity of the Kakapel inselberg (top) where a grinding stone (bottom) was recovered (bottom); (b) Kakoli Hills, a rockshelter with rock art (indicated by grey box, top), depicting four white spirals (bottom); and (c) decorated pottery found in surface scatters from the rockshelters.

**Fig 11:**
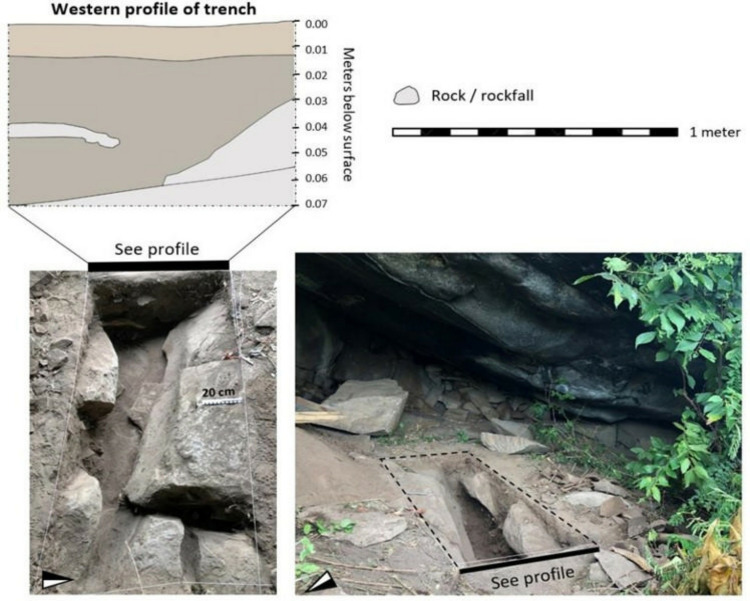
Western profile of the Kakoli Hills trench with photographs of the excavation.

Each rockshelter contained surface scatters of pottery fragments. Half of these scatters (N = 7) preserved ceramic decorations typical of the LIA styles documented elsewhere in the African Great Lakes region in the LIA [75] and frequently recovered from the upper levels of Kakapel (Fig 10–11). In addition to pottery, grinding stones were found on the surface of seven rockshelters and lithic debitage was present at three other rockshelters. Lithics were manufactured from white vein quartz, a material frequently represented in the Kakapel excavation. Additionally, one of these rockshelters preserved a panel of rock art depicting white spirals (S4 Fig).

Kakoli Hills rockshelter, the site bearing rock art similar to Kakapel, is located part way up a steep hill surrounded by mixed-open forest. The site itself extends approximately 5 x 8 m. A 2 x 1 m trench was initiated at the entrance to the rockshelter (Fig 11). Large piles of rockfall from the granite overhang have accumulated along the back of the shelter and this continued into the trench. Large boulders intruded into the excavation, such that the trench narrowed with depth. Granite bedrock began at a depth of 70 cm. The base of the rock was uneven and maximum depth occurred in the southwest corner of the excavation. The western wall of the trench (Fig 11) was the only profile with a sequence of sediment exposed and not obstructed by rockfall. In total, the Kakoli Hills trench produced 31 lithics, 31 ceramics, and 139 bone fragments. These cultural artifacts accumulated through a fine-grained silt with frequent pockets of pebbles, rocks, and boulders. The color of the sediment transitioned from brown to greyish brown approximately 15 cm below the surface and remained greyish brown down to the base.

Quartz lithics, decorated pottery, and rock paintings identified in our 2020 survey resemble cultural artifacts recovered from LIA levels at Kakapel and the white spirals depicted in Kakapel rock art. These finds suggest that the material culture preserved at Kakapel is broadly representative of other localities in the Chelelemuk Hills. However, the rockshelter at Kakapel is exceptional in both spatial and temporal scale. Sites identified in this survey were considerably smaller than Kakapel and we estimate that they preserve a maximum of 1–2 m of sediment accumulation. Consistent with this observation, the Kakoli Hills test trench reached a maximum depth of 70 cm. The extensive stratigraphic record of subsistence strategies at Kakapel is far beyond the scale of other known rockshelters investigated in this region.

### Material culture

#### Pottery.

Excavations have produced 1281 ceramic sherds, including 132 decorated pieces of which 25 are decorated rims (Table 3). In addition, two semi-complete vessels were recovered in 2020. While the overall sample size is small, the assemblage includes several styles and decorative motifs diagnostic to different time periods.

**Table 3:** Ceramic decoration method by approximate time period at Kakapel Rockshelter.

Estimated date range (cal. BP)	Punctate	Rocker^*^	Incision	Impression	Roulette
9400−7000	2	6		4	
7000−3900	4	5	8		
2300−1800	1	1	5	2	1
1200−600	3	2	16		7
600−200			6		12

*Includes zig-zag and serrated edge motifs, after Dale (2007).

**Fig 12:**
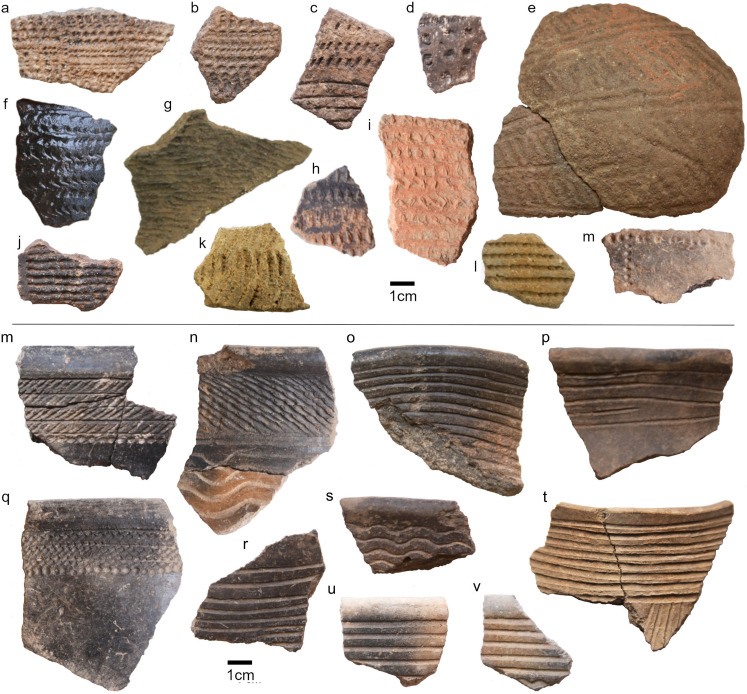
Representative examples of Kansyore (a-m), and Urewe (m-v) pottery from Kakapel Rockshelter. Motifs include punctate (c,d,l,m), rocker motif (k), impression (a,b,f,h,I,j), incised and comb-stamped bands with punctate band (a,e); cord-impressed neck with incised bands (b); linear incised bands (c,d,f-j), and other decoration (g,e).

The earliest ceramics appear in strata dating to 9000–7000 cal. BP, consistent with the earliest dates for pottery used by fisher-foragers along the Lake Victoria shore [30,36,70]. Up to contexts dating to c. 2200 cal. BP, all decorated sherds resemble typical “Kansyore” fisher-forager motifs featuring incisions, various styles of rocker-stamp decoration, and both ovoid and round punctates. Several specimens demonstrate the lattice-like motifs documented from early Kansyore strata at Usenge I and Wadh Lang’o [31](Fig 12 a,b,j,l).

All decorative styles appear to be present from the earliest to latest Kansyore contexts, however; short diagonal incised dashes (below the rim) are only common in the very earliest contexts, and while rocker-stamp and lattice motifs diminish through the sequence, long vertical incisions become more common in later Kansyore contexts (Fig 12c). The few rims from Kansyore levels are flat which may support general patterns of open-bowl Kansyore vessels, however, there are too few large sherds or rims to speculate extensively on vessel form.

A small area in the very back of the rockshelter preserves a discrete “pocket” of ceramics with diagnostic Urewe motifs and forms (visible in Fig 8). Unlike earlier horizons, almost all of the decoration of these ceramics occurs near the rim and consists of incised banding or rows of cross-hatching (Fig 12). Two sherds feature a band of impressed punctates below cross-hatching bands (Fig 12 m,q), and one exhibits a distinctive roulette band with a pendant-like incised motif below it (Fig 12n). All of these features are typical of Urewe ceramics elsewhere in the Lake Victoria Basin [19,88,89].

Several rims are also characteristically beveled, with a 1:1 ratio of rims with flat profiles suggesting open bowls to rims from everted-neck jars [19,65]. There is only one fragment from the small Urewe-bearing area that had a shallow characteristic “dimple.” Small sample size again limits its contribution to discussions over variation in form and function [65,90]. A few sherds exhibit more simplistic and/or less precise versions of the motif that could possibly reflect the so-called “transitional Urewe” or “Lutoboka Group” [19,91], though this attribution is tenuous.

Overlaying contexts dating from 928–685 cal. BP exhibit a technological transition toward pottery with pronounced external or lipped rims with roulette decorations stylistically similar to those documented from western Uganda to central Kenya at this time [75–78,92–94]. There is no evidence for Entebbe traditions. Two roulette forms are present: twisted-string roulette (*torsadée*) and knotted-cord (*nouée)* consistent with LIA styles documented around the African Great Lakes region for this period [75,95].

**Fig 13:**
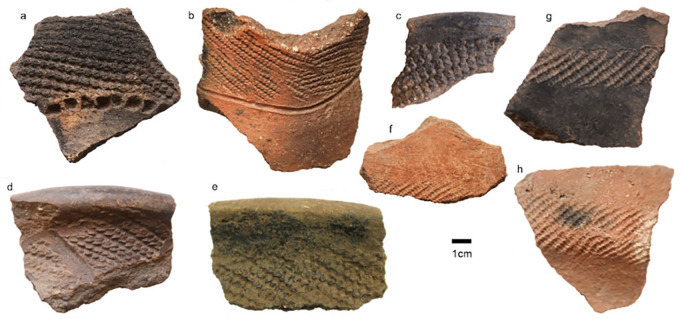
Roulette period pottery: *nouée (a-e), torsadée* (f-h).

The knotted-cord decorated sherds (Fig 13 f–h) show the same diagonal roulette band around the middle of the vessel, and most examples come from two very discrete contexts. One is a large broken *in situ* vessel in a semi-void space bisected by the northernmost extent of the 2020 extension to Trench III, and the other is an accumulation of sherds and large animal bones tucked into a crevice formed by rock spall at the very back of the shelter. Rims of this type are flat and most have wide incisions along the rim, a motif again reported from Iron Age contexts from western Uganda ( [26] to the Central Rift Valley [96,97]. The remainder of the roulette-decorated pottery is a knotted-cord type represented mostly by jars with sharply flared rims. These are found throughout all contexts dated from 900−200 cal. BP. Specimens of this broad category on and very near the surface bear a strong resemblance to ethnohistoric Teso/Luo styles of pottery.

#### Lithics.

The lithic assemblage from Kakapel includes ~11,800 pieces of chipped stone weighing 16.64 kg in total. A subset of this material has been analyzed and is summarized in Table 4. [60] Lithic artifacts are present across all contexts and horizons. The uppermost excavated contexts associated with LIA materials typically have less than 40 chipped stone fragments per 0.1 cubic meter (i.e., per 10 cm level of a 1x1 m unit). The density of lithic artifacts increases markedly with depth, with lower horizons associated with Kansyore fisher-forager pottery containing 100–500 stone tool fragments per 0.1 cubic meter (measured across Trenches II-IV only). While quartz assemblages are well documented for the regions LSA “Kansyore” sites, there have been few discussions concerning continued reliance on stone tool production into the Iron Age. This is, however, not surprising given documentation of stone tool use and particularly production of backed geometrics among ethnohistoric communities in Africa [98]. Indeed, backed crescents are the most common tool form from all horizons.

**Table 4:** Summary of analyzed chipped stone artifact types by archaeological phase at Kakapel Rockshelter.

		*Later Stone Age*		*Iron Age*	
Estimated date range (cal. BP)		9400−7000	7000−3900	2300−1800	1200−600
**Cores**	Hierarchical	5	9		6
	Non-hierarchical	17	11	1	19
	Bipolar	6	8	2	11
**Retouched**	Backed	5	4	2	10
	Scrapers	5	4	0	4
	Other retouched	1	8	2	5
**Flakes**	Complete flakes	47	53	10	69
	Debris	1638	519	403	1968
	*Total weight (g)*	*3483*	*2317*	*980*	*3700*

While the coarse surfaces and high rate of fracture exhibited by vein quartz makes technological comparisons difficult, some differences between LSA and LIA quartz technology are apparent. A higher proportion of LSA cores demonstrate a prepared hierarchical morphology for serial production of elongate flakes from a single platform, whereas LIA cores are more often non-hierarchical expedient forms like radial cores and multi-platform polyhedrons (Table 4). Outside of the shared abundance of backed geometrics (typically crescents) in the LIA and LSA, LSA horizons yielded more shaped tools with more diverse morphologies than the simple forms in the Iron Age (particularly the LIA). This is most evident in the category of scrapers with LSA strata yielding endscrapers, notches, thumbnail scrapers and side-scrapers, while LIA forms are typically roughly shaped convex side-scrapers. These observations are consistent with LSA toolkits oriented around higher mobility, curated, strategies and more opportunistic and expedient lithic reduction typical of more settled communities in the Iron Age.

Raw material diversity is another factor where differences between the LSA and Iron Age are apparent. The vast majority (~98%) of the lithic assemblage is cobble-derived vein quartz across all contexts with a small proportion of obsidian likely from the Central Rift Valley and very low frequencies of cherts, chalcedonies, and coarse volcanics (Table 5). Obsidian is more prevalent from the early Holocene to Early Iron Age (1.9-2.6%) with a considerable decrease in the Later Iron Age (.04%). Most of the obsidian in Iron Age are flake and bipolar core fragments, whereas a high proportion (20%) of the obsidian artifacts from LSA strata are retouched pieces, including 11 backed crescents, two scraper fragments, and three small tabular bipolar cores. Obsidian backed crescents vary markedly in size, between 1.5–4 cm in length. A single large (6.6 cm maximum length) obsidian blade broken into two pieces was recovered in upper LSA Kansyore horizons in Trench III at the very back of the shelter. Higher frequencies of obsidian in earlier periods are likely due to the existence of well documented long-distance exchange networks moving obsidian from the Central Rift to the Lake Victoria Basin until the Iron Age [60].

**Table 5:** Raw material variability for different archaeological phases at Kakapel Rockshelter. Table includes only fragments over 1 mm.

Estimated date range (cal. BP)	Quartz	Volcanics	CCS	Obsidian
9400−7000	1682	2	5	35
7000−3900	599	0	1	16
2300−1800	407	1	4	8
1200−600	2071	1	11	9

#### Bone and iron tools.

**Fig 14:**
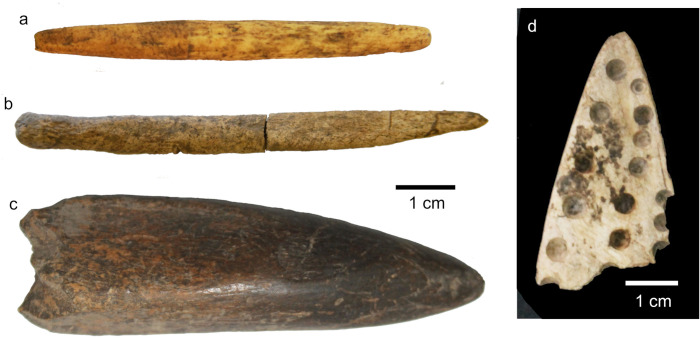
Worked bone artifacts from Kakapel Rockshelter; Shaped points from Early-to-Mid Holocene levels (a-b); robust bone point from Later Iron Age levels (c); Pointed and polished fragment with partial drill holes from Late Iron Age levels (d).

Four bone tools were recovered from excavations (Fig 14). Two were found in association LSA lithic material and Kansyore type ceramics in Trench III contexts overlaying those dates to c. 7300 cal. BP (and so possibly slightly younger). One of these artifacts is a carefully shaped bipoint (Fig 14a) which exhibits wear along two-thirds of its length indicating that this section was covered by a haft. This morphology is consistent with projectile points or link shafts from southern regions of Africa [99,100]. The other (Fig 14b), is a slightly weathered unipoint which may have been used for piercing activities. LIA contexts dating to between c. 1200–975 cal. BP also in Trench III produced one 9 cm long bone shaft fragment ground into a point at one end with rounded edges, probably during use or for a specific function like leather working or pottery shaping. Another worked piece from upper LIA horizons dating to 732–898 cal. BP (Wk-48699, 887 ± 18 rcybp) was a flat bone with 14 circular ~1 mm wide partial drill holes on one side and polishing on the opposite. The latter artifact resembles a bow-drill block, but its function is unclear. While there is an apparent difference in the smaller bone points from LSA contexts and the more diverse objects from the LIA, the small sample size makes it difficult to determine how much bone tool technology was changing over time.

**Fig 15:**
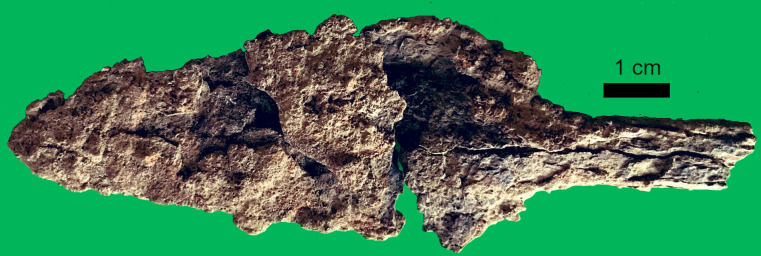
Iron spear point from contexts dated to 900-960 cal. BP.

Despite the deep Iron Age sequence, only a few fragments of iron have been recovered from Kakapel Rockshelter. Most of this collection consists of flat fragments of iron, several small fragments of ironworking slag, and a single iron spear point (Fig 15). All iron artifacts were found in Trench III LIA contexts dating to after c. 1000 cal BP with the spear point coming from contexts dating to around 911–752 cal. BP (920 ± 20 rcybp, OS-167170).

#### Beads.

**Fig 16:**
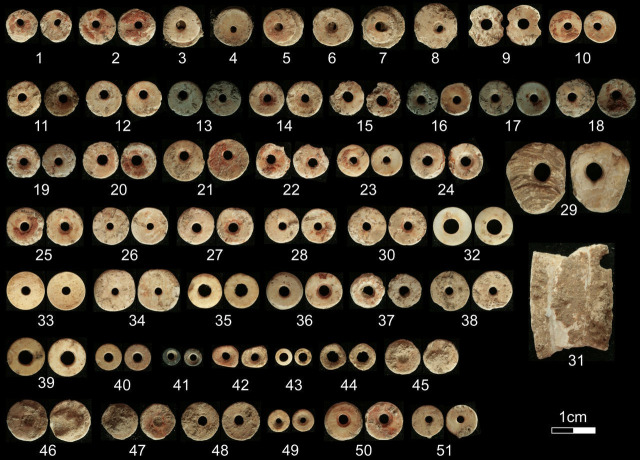
Variation in disk beads from Kakapel Rockshelter. All are terrestrial shell except for 29, 31, which are likely made from Nile oyster shell. Note the red-ochre residue visible on 1, 2, 10, 19-22, 25, 27, 28, 47, 50.

In total, 60 finished shell disc beads were recovered, including 10 on ostrich egg-shell (OES), 47 on land snail-shell (LSS), and three on aquatic-species shell; most likely Nile oyster (*Etheria elliptica*) (Fig 16). There is no evidence for on-site bead manufacture. Almost all (82%) of the bead artifacts were recovered from stratigraphically secure Kansyore contexts.

Fifty-three percent of all beads were directly associated with Burial 1, with many recovered around the individual’s legs and all these beads were produced on land snail-shell. The LSS beads from Burial 1 are larger with smaller apertures than the rest of the Kakapel assemblage. Analysis of the Burial 1 bead apertures indicates at least six were drilled starting from the outer shell surface, and 10 bear signs of drilling with a hand-turned implement rather than a hafted drill bit [101]. Not only are these unusual traits among LSS and OES bead assemblages from other sites [102] but they contrast with the remaining Kakapel beads (one drilled from the outer surface, no evidence of hand-turned drilling), and highlight a potentially distinctive cultural style of the Burial 1 bead assemblage. Beyond Kakapel, beads associated with Burial 1 are also slightly larger (6.06-10.21 mm, avg 8.29 mm) than typical assemblages from eastern African Holocene forager sites (4.73-10.07 mm [avg 6.65 mm]) [103].

Bead raw materials increase in variety through time at Kakapel, with diversity that coincides with the onset of the LIA. Beads associated with Burial 1 are almost exclusively made from locally abundant land snail shell. By raw material, 67% of OES beads and all aquatic shell beads were from LIA contexts. While the Iron Age sample is small, beads from this period tentatively appear smaller than those from earlier Kansyore contexts (Mann Whitney U: 73.5, z = 2.84, p = .0036, see also S1 Table). Non-disk beads are represented only by three specimens: a pierced cowrie shell from LIA contexts (c. 900 cal. BP), a bone tube-bead within the pit-fill of LSA Burial 1, and an ovoid bead with two centrally aligned perforations made from a bone shaft fragment.

All but 15 disk beads exhibit red ochre residues. All disc beads found associated with Burial 1 display ochre. Notably, the red ochre residues on the beads found near the burial are extremely thick, indicating either that the beads were deliberately coated with ochre prior to or in association with the interment, or, that the beads gathered the residue incidentally through extended contact with painted skin during their use life. The two oyster artifacts have no trace of red ochre.

### Biological remains

#### Fauna.

Excavations produced 6,902 g of faunal remains which were described with both conventional zooarchaeological methods and ZooMS analysis. Details of the methods, faunal identifications, and ZooMS results are published elsewhere [18] and are summarized here.

Faunal remains from LSA contexts include only wild species, with high representations of mid-sized bovids such as reedbuck (*Redunca* sp.) and bushbuck (*Tragelaphus scriptus)* and hartebeest (*Alcelaphsus buselaphus*). ZooMS supports common use of diverse Alcelaphines, Reduncines, and Tragelaphines, and also exploitation of wild African buffalo (*Syncerus cafer*) [18]. Reptiles, tortoises, and rodents were also present in lower proportions and while these can be hunted species, it is not clear what proportion of these specimens reflect intentional exploitation versus natural rockshelter taphonomy. Predator species were represented by only two finds from Trench IV: a single leopard molar and one fragment of ZooMS-identified hyenid/felid/mustelid bone.

Lower frequencies of faunal remains were present in the EIA contexts, but these continued to be dominated by wild species and particularly mid-sized mammals, and smaller reptiles and rodents. The only exception is the identification (confirmed by ZooMS analysis) of a single domesticated cattle bone directly dated to 2,331‒2,155 cal. BP (OxA-40173, 2235 ± 20 rcybp). This bone fragment was found directly outside of and on the same horizon as a clay-lined hearth feature in Trench III where we recovered the early cowpea with a similar date (OS-168214, 2280 ± 20 rcybp). Aside from being the earliest directly dated evidence for domesticated fauna in the Lake Victoria Basin, the finding supports EIA groups having nearly simultaneous access to crops and livestock. Use of domesticated species increases only slightly into the LIA where cattle and goat are both present. Otherwise, LIA fauna continue to reflect the same savanna bovid species and smaller rodent and reptile types as the LSA horizons [18].

#### Human remains, bioarchaeology, and archaeogenetics.

**Fig 17:**
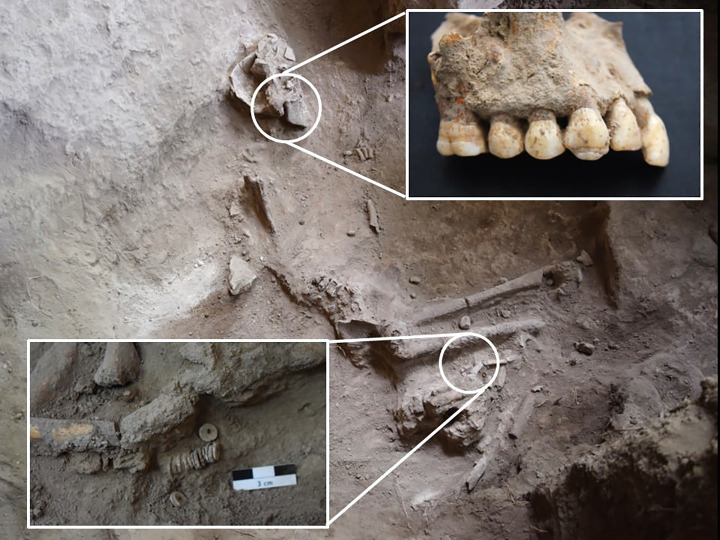
Burial 1 at Kakapel Rockhselter associated with Kansyore material culture; insets highlight dental hypoplasias and the row of shell beads near the right leg.

A minimum of nine individuals are represented in the human remains excavated from Kakapel Rockshelter. The sample includes three individuals recovered *in situ* from primary interments beneath the rockshelter overhang (Burials 1–3), a partial infant cremation (Burial 4), and at least five other individuals represented by isolated human remains. Burials 1 and 2 were encountered in 2015 and excavated in 2018, while Burials 3 and 4 were encountered and excavated in 2020. The three individuals in primary burials were flexed and lying on their left side, but orientation and positioning of hands and legs varied. Burial 1 was associated with shell beads, some of which remained aligned as when strung (Fig 17), while the other two burials did not have clearly associated mortuary goods. Skeletal preservation was excellent for the LIA burials (Burials 2 and 3) while Burial 1 suffered more taphonomic damage to the postcranial skeleton. The crania of Burials 1 and 2 both rested near large boulders. Burial 4 included infant remains found during flotation of a large and heavily compacted burnt feature. Bones of this individual are highly fragmented, except for the mental eminence and left mandible, and left tibia. All elements are blackened from burning, to varying degrees, suggesting these remains were still fleshed when subjected to burning at a relatively low temperature [104].

Burial 1 dates to 3900 cal. BP (3584 ± 28 rcybp, SUERC-86057) and the individual was most likely 20–30 years at the time of death, based on complete eruption of the third molars, complete but visible line of fusion on the proximal femur, and morphology of the pubic symphyseal faces and auricular surfaces of the pelvis [105,106]. Based on skeletal and genetic evidence, this individual was male and suffered sustained childhood stress or illness as evidenced by multiple linear enamel hypoplasias on both the anterior and posterior teeth (Fig 17). This individual was directly associated with a string of shell beads near the legs and contexts surrounding the burial yielded lithics and Kansyore ceramic sherds.

Burials from LIA contexts included an adult female (Burial 2), a juvenile (Burial 3), and an infant (Burial 4). The Burial 2 individual was most likely in her 40s when she died based on dental eruption, morphology of the pubic symphysis and auricular surfaces of the pelvis, and degeneration of the first rib [105–107]. The Burial 3 individual is estimated to be around 10 years of age at the time of death, based on dental eruption, epiphyseal fusion, and long bone length [108–110]. The infant in Burial 4 is estimated to be 6 months (+/- 3mos) at the time of death based on development of enamel crowns and long bone length [109,110]. Pathology for the Iron Age individuals includes skeletal asymmetries (Burial 2), indications of infection (Burial 2 and 3), and linear enamel hypoplasia (Burial 3). Trauma includes perimortem compression fractures to the cervical spine on Burial 3. Stature and mass estimates for Burial 2 are based on the complete right humerus, radius, ulna, femur (including head), and fibula, and suggested a height of 153.5 ± 4.25 cm and 158.0 ± 5.05 cm and body mass between 34–41 kg [111–113].

At least five additional individuals are represented by isolated remains. These include two isolated teeth from Trench II (one sampled for aDNA) recovered in 2018, and a partial foot, finger bone, and isolated tooth recovered from Trench III. Isolated remains from the 2020 excavations represent a minimum of three additional individuals. This includes two teeth and a metacarpal from Trench II, and three teeth, two hand phalanges, one foot phalanx, and one vertebra from Trench III. It is not clear what behavioral or taphonomic processes led to the presence of so many partial remains; however, at least some of the material may be from burials disturbed by later occupational phases.

Ancient DNA was successfully extracted from three individuals recovered during the 2018 season: the left petrous from Burial 1, the right maxillary central incisor from Burial 2, and a duplicate right maxillary lateral incisor found in the strata into which the Burial 2 pit intruded. Genomic analysis revealed these individuals represent three distinct occupations with dramatically different ancestry profiles [13]. The individual from Burial 1 broadly shares ancestry with other ancient foragers in eastern Africa, with a component similar to recent Mbuti hunter-gatherers [13]. Both the Burial 2 individual and the person represented by the isolated tooth show close genetic affinities to Dinka and other Nilotic-speaking groups. The ~ 900 BP individual represented by the tooth also shares ancestry with northeastern Africa/Levantine groups, suggesting admixture between Pastoral Neolithic-related herders and incoming Nilotic agropastoralists sometime in the Iron Age. This individual is temporally associated with most of the evidence for finger-millet and sorghum agriculture at Kakapel. The use of Kakapel rockshelter by at least three genetically-distinct groups over its 9000-year sequence is consistent with archaeological and genetic evidence for multiple population movements of diverse food producers during the Holocene.

## Discussion

### Occupational history

Kakapel rockshelter, with its multi-phase sequence, captures occupation by a series of culturally, economically, and genetically diverse communities in the Lake Victoria region. Radiocarbon dates spanning Early-to-Late Holocene hunter-gatherer periods, different ceramic phases of the Iron Age, and pre-colonial periods indicate the site, and the hills surrounding the Lake Victoria Basin and its drainages, played a consistently important role in diverse subsistence economies through time.

The earliest detectable use of the rockshelter was by hunter-gatherer societies in the late 10^th^ millennium BP, as indicated by two radiocarbon dates in association with lithics, bones, and ceramics from separate parts of the site. These dates are consistent with early dates for the pottery at Siror and Gogo Falls [36], though sherds from early levels are small, weathered, and too few are decorated to definitively attribute them to the “Kansyore” tradition. Regardless of affiliation, these are among the earliest securely dated examples of ceramic production and use in eastern Africa.

Ceramics are slightly more abundant and exhibit typical early Kansyore motifs in contexts dated to 7426–6900 cal. BP. These dates are associated with a more substantial occupation in and around the rockshelter, as evidenced by the dense artifact horizon dating to this period in Trenches II and IV and in the lower portions of Trench III. Lithics are most common in this period, and recovery of pitted anvils and hammerstones reflects frequent lithic reduction taking place at the site. In combination with bone tools and shell beads from these levels, the package of material culture accompanying Kansyore ceramics is like that reported from other sites dated to the same period around the Winam Gulf of western Lake Victoria [31,36–38]. Artifact density in these contexts is also at least as high as other Holocene fisher-forager sites in the region and so it does not appear that Kakapel was a peripheral occupation relative to the lakeshore sites. There is no evidence of long-term occupation at Kakapel (such as houses), and the high quantities of lithic and faunal remains are more likely a product of multiple repeated short-term or seasonal occupations.

It is not yet clear if people continued to visit Kakapel during the “Middle Kansyore” hiatus period. Like many other parts of the Lake Victoria Basin, it appears that there is an occupational gap from the late 7^th^ to 5^th^ millennium BP [41]. A human burial dated to 3976–3777 cal. BP indicates that LSA foragers were using the rockshelter by that time, but at far lower intensity than is evident for in the Early Holocene. Ancient DNA recovered from this individual demonstrates that forager populations here shared ancestry with other hunter-gatherer groups across eastern Africa but also with recent Mbuti populations [13]. This result indicates that at least by the Mid-to-Late Holocene, interaction spheres included diverse groups from the Great Lakes to the eastern Congo Rainforest (see also Schmidt et al., 2024).

While not present in the 2016–2018 excavations, extensions to Trench III in 2020 detected a discrete feature-dense area (under 1 x 2 m) against the back wall of the shelter with distinctive EIA Urewe ceramics. These deposits were stratified in the trench profile, and they may have extended further from the shelter wall before being disrupted by a large LIA pit feature. Dates across the Urewe-bearing features yielded dates from c. 2,300−1,700 cal. BP consistent with EIA occurrences around Lake Victoria [19,20,47,70]. Despite the several hundred year range of dates, the relatively limited horizontal extent of these strata suggest they were formed by few, short-term, uses of the site. These features are important in yielding early evidence of domesticated cowpea and domesticated cattle reflecting an economy with agropastoral components in association with Urewe ware by 2,300 cal. BP [18],

Radiocarbon dates from discrete features across the excavated portions of Kakapel attest to a period of more concentrated occupation from 928–685 cal. BP. A horizon rich in Roulette-decorated ceramics occurs across all excavated portions of the site, and this horizon includes several pits, ash-dumps, middens, and hearths. This appears to include both a minor component of earlier *torsadée* roulette motifs immediately overlaying Urewe contexts, and more substantial period of *nouée* roulette motifs that are common across the northern and western Lake Victoria Basin [75]. LIA pottery and grinding stones are also the most abundant form of material culture detected in surrounding rockshelters.

The uppermost portions of the Kakapel Rockshelter stratigraphy show that use of the area by agropastoralists continued into recent centuries. This situation is especially evident in the historic forms of Roulette pottery on the surface, and two burials that can be attributed to the last 600 years. Findings are consistent with local oral histories that attest to the presence of large homesteads or small villages once placed against the rockshelter. It is clear from archaeogenetic evidence that a third detectable population is responsible for these occupations, entirely consistent with the initial movement of Nilotic-speakers into the region from the Ugandan highlands [13]. It is not yet clear when the Teso speakers who currently occupy the region arrived, but the last few hundred years saw a clear shift toward more special-purpose use of the site. For example, the larger burning feature with cow bones in Trench V is similar to ethnohistoric meat-feasting deposits [114] which would be consistent with oral history of the site having a more ceremonial function in recent centuries.

### Economic transitions at Kakapel Rockshelter

Archaeological excavations, digital mapping, recovery of abundant plant remains, and multi-disciplinary analyses have made Kakapel Rockshelter a high-resolution data point that impacts models for food-system change in the Lake Victoria region of eastern Africa through the Holocene. Combining the archaeological data discussed here with subsistence data from Kakapel [18] allows for a more holistic reconstruction of economic change at the site.

Discovery of substantial Early Holocene forager occupations with Kansyore type pottery adds to a growing recognition that lifeways across the Great Lakes were more diverse than the typical construction of “Kansyore fisher-foragers” [26]. LSA occupations at Kakapel are not linked to a fishing adaptation, but rather demonstrate a strong emphasis on terrestrial hunting of mid-sized bovids [18]. While snakes, reptiles, and rodents may be partially taphonomic, the diverse array of small fauna in forager levels may indicate broad spectrum hunting and trapping. Hunting may have become more important in later periods at lacustrine sites, but they were a prominent strategy at Kakapel from c. 9200 cal. BP onward [31]. Bone tools may reflect technological dimensions for exploiting a broad resource base. Recovery of large quantities of wild plant remains including edible species in Early to Middle Holocene contexts hints at the kind of plant gathering practices that were also likely to have been critical for forager livelihoods but which have been rarely discussed by archaeologists [18].

Further research is needed to determine the regularity and duration of forager occupations at Kakapel and the Chelelemuk Hills broadly. Dense artifact horizons including features, bone middens, evidence for diverse wild resources, and a burial are not consistent with expectations that Kakapel was only a peripheral hunting camp within seasonal rounds otherwise focused on lacustrine and riverine resources [30,31,38,41]. Rather than a logistical stop-over, Kakapel may have been a medium-term or seasonal base camp [115,116] for foragers whose subsistence economy differed markedly from Kansyore-producers elsewhere in the Lake Basin. This does not mean these populations were isolated. Imported obsidian across all layers indicates ongoing contact and down-the-line exchange reaching into the Rift Valley to the east, while archaeogenetic evidence reflects previous admixture with populations in the western Congo Basin. The geographic range and distributions of Kansyore pottery may indicate that “Kansyore” may, in part, represent a widespread and sustained interaction sphere connecting diverse communities [26].

Findings from Kakapel also inform models for the spread of food production in eastern Africa. Detection of domesticated cowpea (a likely West African domesticate) in direct association with diagnostic Urewe pottery supports a connection between early agriculture, EIA material traditions, and the Bantu Expansion [18,20]. These relationships are likely complex and non-uniform. The absence of pearl millet from extensive flotation at Kakapel might indicate these crops spread spatially and/temporally separately and were not part of a “package” of early farming expansion. EIA populations did, however, have access to domesticated cattle, marking the beginning of livestock keeping in lifeways around Lake Victoria before dedicated pastoralist sites are detected there [18].

Thin and spatially restricted EIA deposits were formed by smaller-scale and possibly shorter occupations than either earlier or later periods. Rather than settled farming villages or the larger Urewe sites documented around Lake Victoria, Kakapel appears to capture smaller and more mobile dimensions of EIA communities [4,26,36,70]. Given the early direct dates for Urewe at Kakapel (c. 2,300 cal. BP), further research may reveal that initial ephemeral expansions preceded establishment of longer-term and larger EIA habitation sites. Most importantly, the findings from Kakapel draw attention to the period from c.2,500−1,700 cal. BP as a time of important cultural and economic transformations in eastern Africa.

The LIA deposits at Kakapel are marked by a noted increase in occupational intensity as evidenced by dense material culture, numerous overlapping features, burnt-clay derived from structures, grinding stones, and multiple burials. Identification of LIA material in rockshelter sites surrounding Kakapel suggests this use was part of a broader pattern of increased settlement of the Chelelemuk Hills after c. 1,200 cal. BP. Greater regional settlement is coincident with new populations spreading into the region [11,13] and the appearance of an agropastoral economy focused on sorghum and finger millet [18]. Settlement during the LIA appears to have occurred in at least three archaeological phases. The first is marked by high quantities of finger millet and *torsadée* decorated LIA ceramics with flat, impressed, rims from 1,200−900 cal. BP. From 900−300 cal. BP sorghum and pea (*Pisum*) become more frequent in the assemblages [18] along with pottery with diverse *nouée* Roulette motifs. This is followed by recent expansions of Nilotic speaking peoples from Uganda, including the Teso-speaking peoples who occupy the regional currently.

Increased human occupation along the inland lower slopes of Mt. Elgon during this time was certainly aided by the adoption of grains like finger millet and sorghum that are well adapted to highland environments and so could support large populations in this region. High agricultural potential may have been especially important at this time due to climatic instability across the Great Lakes [46]. Agropastoralist populations were already larger than in previous periods and could have been drawn to the Chelelemuk Hills as a relative climatic refugium. It is therefore a combination of environmental pressure, social change associated with the rise of Bantu polities in Uganda, and the arrival of new agricultural systems that led to more focused occupation of Kakapel Rockshelter and the surrounding Chelemuk Hills.

## Conclusions

Collaborative field investigations around Mt. Elgon and subsequent excavations and multi-disciplinary analyses at Kakapel Rockshelter and across the surrounding region have revealed a record of shifting populations and shifting subsistence practices in the Lake Victoria Basin of eastern Africa that spans the last 9,000 years. The Kakapel Rockshelter is a rare example of a site preserving such a deep-time multi-phase record with Early and Late Kansyore Early Iron Age, LIA, and proto-historic occupations in the region. Kakapel is thus far the best dated Holocene site in the region and has produced one of the largest archaeobotanical assemblages in eastern Africa including the only evidence for domesticated grains around Lake Victoria [18]. Combined with analyses of faunal remains, material culture, and recovery of human remains and ancient DNA, these data provide critical new insights into trajectories of food production in the Lake Victoria Basin, and across eastern Africa broadly.

Evidence from Kakapel Rockshelter supports a regionally heterogeneous and complex pattern of food production in the Lake Victoria Basin. Hunting patterns established in the early Holocene continued to characterize strategies through the Iron Age and into pre-colonial periods. It does not appear that communities living at Kakapel ever developed a strong reliance on domesticated animals [18]. This contrasts with the more livestock-focused economies that appear in Pastoral Neolithic sites elsewhere around Lake Victoria after c. 1900 cal. BP [4,36,38]. Iron Age horizons reveal the development of an agricultural economy by around 1200 cal. BP that was centered on eastern African finger millet, with a small component of sorghum as well as assorted wild plants.

The Kakapel findings are incompatible with any models that propose a single process, or series of independent expansions, for the spread of different food-producing strategies. While domesticated plants first appear in association with Urewe style ceramics by c. 2,400 cal. BP, this arrival does not manifest as a complete “package” of crops utilized historically in the region. Instead, a succession of incoming populations exhibited local agency in the degree to which they adopted long-standing use of wild plant and animal resources or integrated specific crops into agricultural systems. It is likely that such diversity characterizes different parts of the Lake Victoria region. Specific population histories and local ecological/climatic conditions would have played major roles in influencing local trajectories. As studies of food production in the region expand, it will be critical to begin investigating the degree to which local ecological knowledge and specific domesticates were transmitted between diverse Holocene communities, and how these processes shaped the forms of food production that spread from the African Great Lakes region further east and south across the continent.

Research at Kakapel Rockshelter also demonstrates the potential for applying multi-disciplinary research that integrates emerging techniques in archaeological science. First and foremost, the recovery of abundant archaeobotanical remains dispels the long-repeated assertion that plant remains do not preserve in western Kenya. Combining high-resolution mapping, archaeobotanical and zooarchaeological analyses, focused bioarchaeology and ancient DNA techniques, and community oral histories allow for more holistic reconstructions of population and subsistence at the site, and for the region broadly. These new insights allow archaeologists to test proposed models for the spread of food production, regional settlement patterns, and hopefully, to generate new ones that better capture the heterogeneous nature of Holocene transitions in eastern Africa.

## Supporting information

S1 Text Descriptions of archaeological contexts defined during excavations. (DOCX)

S1 Fig Surface topography of the carbonate feature in Trench III showing slope of the feature that cuts through multiple occupational horizons. (TIF)

S2 Fig Portions of the carbonate feature as it appears in upper LIA areas (a), and overlying LSA strata (c), with image of a hearth on top of the carbonate (c). (TIF)

S3 Fig Detail of the smaller Kakapel Rockshelter II area (a) and stratigraphy (b). (TIF)

S1 Table Measurements and attributes of the shell bead assemblage from Kakapel Rockshelter. (XLSX)
