# Supplementary material for: Cultural, economic, and settlement shifts over the last 9,000 years at Kakapel Rockshelter, Western Kenya
Source: PLoS One. 2025 Aug 20;20(8):e0328805. doi: 10.1371/journal.pone.0328805 (PMC12367187; doi:10.1371/journal.pone.0328805)
Supplement: S1 Text — (DOCX) [file pone.0328805.s001.docx]

**Supplemental Text 1**

**Cultural, economic, and settlement shifts over the last 9,000 years at Kakapel Rockshelter, western Kenya**

Steven T. Goldstein^1*^, Natalie G. Mueller^2^, Emma Finestone^3^, Elizabeth A. Sawchuk^3,4^, Sara Juengst^5^, Anthony Odera Otwani^6^, Jennifer Miller^7^, Michelle C. Langley^8^, Ricardo Fernandes^9,10,11,12^, Axel Steinhof^13^, Victor Iminjili^9^, Anneke Janzen^14^, Christine Ogola^15^, Christine Chepkorir^1^, Emmanuel Ndiema^16^, Michael Petraglia^8,17,18^, Nicole Boivin^9,18,19^

**Excavation units and defined archaeological contexts**

Context numbers and descriptions are below, for comparison with radiocarbon dates reported in Table 1. Single digit context numbers relate to excavations in 2018, and three digit numbers relate to contexts excavated in 2020. Whole numbers denote contexts whereas decimals refer to arbitrary subdivisions of contexts when they continue beyond 10 cm increments in depth with no clear change in material or matrix (e.g. 107.1 denotes continuation of 107.1 beyond 10cm, 107.2 is a second subdivision of 107 matrix continuing past 20 cm).

**Trench II**

Context 1: This consisted of Burial 2 and the surrounding sediment in the northern portions of Trench II.

Context 2/101: Soft ashy sediment below Context 1 in the western half of the 1x2. This context produced seeds of domesticated *Sorghum bicolor*.

Context 3/102: More compacted sediment in eastern half of the trench, equal to Context 2.

Context 4: Mottled brownish grey sediment below Contexts 2 and 3, contained prehistoric pottery and lithic artifacts. This context produced seeds of domesticated *Sorghum bicolor*.

Context 5: Ashy layer with abundant charcoal below Context 4, spanned the entire 1x2 portion of the trench. Parts of Context 5 extend deeper into a horizon that was equal to Contexts 6,7, and 8. This was labeled Context 5.1.

Context 6: Large termite disturbance in southeastern portion of the excavation unit, directly interfaces with Context 7.

Context 7: Compacted feature, possibly a habitation surface. The edges of the feature were heavily reworked, especially where it interfaces with the insect disturbance in Context 6. Context 7 features heavy rooting.

Context 8: This is a darkened hearth feature in the southwestern portion of the 1x2 m portion of the trench, on the same horizon as Context 5.1, 6, and 7. This feature was bisected by the portion of the 2015 excavations that continued to the eroding bedrock.

Context 5.2: Ashy sediment similar to Context 5 and 5.1 that continues below the other Contexts in the western half of the unit. On the same elevation as Context 11 in the eastern portion of the unit.

Contest 9: Large, possibly rock-lined hearth feature within Context 10. Contexts 9 and 10 are below Context 5.2

Context 10: Interface with more red and slightly more compacted sediment. Larger rocks present in this level.

Context 11: Hardened and compacted sediment below, and related to, the possible floor feature in Context 7.

Context 12: Below Context 10 was a softer sediment, less mottled and with very little charcoal but still pale red sediment with frequent granitic inclusions. Context 12 appeared to be grading more into eroding bedrock. Very little material culture was found in this context. Context 12 was slanted steeply upward to the east (toward the rockshelter) following the shape of eroded bedrock. This was visible in the profile.

Context 13: Harder but discontinuous layer below Context 11, cut into by Context 12. Context 13 is likely related to Context 11, but angled under Context 12 due to the steep angle of these deposits.

Context 14: Orange-brown sediment, extremely compacted and very high rate of granitic cobbles and pebbles. This context is nearly sterile after 3 spits of 10cm, and appears to be eroding bedrock material. Excavates ended at this context.

Context 104: Black semicircular bowl-shaped profile feature. At 5cm depth encountered lens of charcoal and ash that ended the likely hearth feature.

Context 105: Dark brown to black charcoal rich feature at the same plane but distinct (40 cm away) from 104. The shape of the feature was irregular and only ~2cm in depth, possibly a secondary dump or disturbed cooking feature.

Context 106-106.1: Mottled brownish grey sediment, similar to context 4, below plane of features 104-105.

Context 107: Darkened charcoal rich feature extended across almost the entire excavation unit below 106.1.

Context 108: Start of yellow-brown sediments with more frequent rockspall inclusions (granules to cobbles).

Context 109: Slightly arbitrary division from 108 with minor change of looser sediment and slightly more reddish yellow coloration.

Context 110: Arbitrary transition as sediment color continues to grade to darker reddish-yellow shades of brown and inclusions from rockspall becoming larger (up to 35cm). Contains dense lithics and Kansyore pottery, and ochre.

Context 111: A small (<30 sq. cm) patch of ashy sediment on the eastern edge of the excavation unit within 110.

Contexts 120: Transition to reddish-brown more compacted sediments (appears the same strata as Context 10), with decreasing artifact density but increasing density of granitic inclusions.

Contexts 121-127: Arbitrary 5cm levels continuing below 120, very gradual change to more orange-brown sediments that grade into eroding regolith at the bottom of the trench, equivalent to Context 14. Sporadic lithic material and charcoal continues through 126, but 127 is entirely sterile.

**Trench III**

Context 1: Context 1 was all sediment in the eastern half of the 2x2 around Context 2. Context 1 wasloose brown sediment with abundant material culture and faunal remains.

Context 2: This is a semi-circular, pale, ashy feature in the northern portion of N17. It appears to be a secondary ash dump with abundant animal bone and other artifacts. This context was about 5cm of white ash with another 5cm of grey ash.

Context 3: This encompassed Burial 1 and the associated sediment that appeared to be within a burial pit. Because the 2015 excavations reached the burial, we were unable to determine if this was a true pit or where the pit began in the unit stratigraphy.

Context 4: All sediment surrounding the Context 3/ Burial 1 context. It featured a high proportion of fauna. It was more compacted than Context 3, otherwise there was no obvious color or matrix difference.

Context 5: Grey ashy feature immediately to the east of Context 3/Burial 1. Appeared to be another secondary ash dump.

Context 6: Beneath Context 2 (still above the burial) was a very compacted surface feature. Immediately below Context 2 in N17 this was flat, however the portion that extended into N16 had broken and was angled at roughly 30 degrees. A large portion of this surface would have extended over Burial 1, however this was removed in the 2015 excavations and so we could not record it here. Another portion of Context 6 was found at an even lower elevation in N15 about 30cm above the knees of Burial 1.

Context 7: Interstitial sediment between Context 6 and surrounding features.

Context 8: Finer, siltier, brown sediment beneath Context 2 and 6 covering most of N17 but extending partially into M17 and N16.

Context 9: Appeared as a circular patch of grey ashy sediment roughly 45-50cm in diameter. All sediment was removed for flotation. Further excavation and bisection of the feature revealed it to be a semi-compacted clay-lined hearth feature surrounded by darkened/burnt sediment.

Context 10: This was the brown silt sediments below Contexts 8 and 1 that covered all of N and M 17. It was very rich in artifacts, including a very large backed obsidian microlith and heavily decorated Kansyore pottery found at 112cm below datum. It is possible that Context 10 marks a shift to an earlier phase of Kansyore occupation.

Context 11: A portion of a compacted surface that had been heavily reworked – possibly related to nearby animal burrowing.

Context 12: Softer sediment below Context 10, contained increasing proportions of granite rockfall of cobble and larger sizes. Sediment was mottled, interspersed with grey ashy patches and very dense fauna. Context 12 appears to sit on top of a very large fragment of rockfall that was angled slightly back toward the shelter abutting a perpendicular upright fragment of rockfall. This created a “bench” with a slight crevice extending under the perpendicular rock. Context 12 appeared to be successive dumps of refuse mixed with incidental accumulations of natural and anthropogenic debris onto this bench. Below Context 12 was a large rock and so this is the end of excavations in the eastern portion of the trench.

Context 13: This describes all sediment on the north side of the large central rock except for Context 11. It contained several large bovid shaft fragments. This context partially overlied the burial context and so was excavated in arbitrary spits until the burial context was fully exposed. At this point enough of Burial 1 could be exposed to determine that the legs of the individual extended westward into what would be unit N15. Units N15 and N14 (a 1x2 extension) were added to fully expose the burial. Five total spits of 10cm were excavated above this portion of Context 6, and were characterized by a mottled brown sediment with minor insect disturbance and rooting. Later Iron Age Roulette phase pottery characterized the upper 20cm of excavation, followed by a transition to lithics and Kansyore ceramic styles. The proportion of clearly prehistoric ceramics increased after 40cm below datum. The last 10cm spit of N15 featured several thin overlapping ashy features that may reflect episodes of hearth use in the area.

Context 14: Surface underlying the burial pit and surrounding sediments. Context 14 was a compacted fine ashy sediment, and had very little material culture. It contained abundant animal bone, but much of this was reptile, cane rat, and hyrax, likely representing natural accumulation under the rockshelter. More and more fragments of rockfall of all sizes were encountered through Context 14.

Context 15: Greyish circular feature in southern part of N16 between two larger rocks, still yielding fauna but few artifacts. This may have beeen in-fill from above into the rock crevice.

Context 16: All sediment across Trench 3 except for the feature described as Context 15. Very rocky, with several slabs of granite rockfall, especially dense in the SW corner. Boulders become more common with depth and increasingly sterile.

Context 17: 10-15cm below Context 16, the sediment became sandier and dominated by large slabs of rockfall. Several cm of large rockfall was removed, but it continued below and could not be excavated. Excavations in this trench ended at this context.

Context 18: Compacted sandy silt surface. Trampled A horizon. Several burrows and other disturbances were visible. This excavation unit was in a more protected area further “back” in the rockshelter and much more rodent activity was visible here.

Context 19: Fine silts with some clay component. Several large burrows were visible and the layer appeared to be mixed. Material culture was primarily historic and Later Iron Age pottery.

Context 20: Upper, middle, and lower portion of a pit or burrow feature visible in the profile, but were only visible as lightly grey coloration during excavations. All sediment wasloose silts, and these divisions may reflect gradual filling in of the pit over time, suggesting it was likely a large natural burrow. Few artifacts were present in the small part of this feature which was in the excavated area.

Context 21: Moderately compacted darker brown silt with common pebbles and granules. Resembles the B horizons elsewhere on the site, but in this trench it was s only evident in units N14 and N15. Artifacts were slightly mixed and include historic and prehistoric pottery. Animal bone and obsidian become more frequent.

Context 22: Equivalent to C.21 in stratigraphy but clearly separate. C22 was very anthropically derived, darker and more organic rich than C.21. Artifacts were more common and it included a dense overlapping hardened carbonate surface feature. This feature was partially broken up and reworked in the sediment around its periphery. These may have derived from successive habitations set against the back of the rockshelter.

Context 23: Underlying C.21 in units N14 and N15 further from the rockshelter. This context consisted of coarser more compacted silt with some clay and sand components. Granules and pebbles were increasingly common.

Context 101-101.2: Surface and sub-surface sediments

Context 102: Interface with lighter brown sediments/

Context 103: Pale grey ashy feature exposed in the north profile of 2018 trench, entire context was taken as a flotation sample.

Context 104: Brownish grey sediment containing slightly higher frequency of bone including disarticulated juvenile human foot bones. Excavations did not identify any a primary burial associated. As excavated, revealed a more defined artifact surface below labeled context 104.1

Context 106: A small grey-white ash feature in the mottled brown-grey sediment of context 105. Likely an Iron Age hearth.

Context 107: Hardened and compacted ash and daga/clay rubble surrounding feature 106. Lower portions that extend into underlying more orange mottled sediments labeled as 107.1.

Context 108: Pale brown sediments surrounding 106 and 107 with higher rate of rock inclusions (pebbles to small cobbles).

Context 109: Irregular extent of paler sediments within 108 extending into 110- no clear variation in inclusions or material culture density.

Context 110-110.4: Below 108 but intersecting with 109 is a brown to orangish brown very compacted sediment.

Context 111: Interstitial soft brown sediments separating 112 from 110.2.

Context 112: Very compacted grey ash feature. Appeared to be rectangular in plan view when first encountered but an additional 1m extension was added to the eastern end of the unit to fully expose it. At full exposure it was a was more oval in shape and extended along the back wall of the shelter- extending over 2m in length and 1m in width. Appears to be a compacted surface- possibly a floor and surrounded by a softer matrix (111). Human deciduous tooth, large faunal bone, and broken in situ pot within this feature.

Context 113-113.1: All sediments below plane of 110.2,111, and 113, more compacted brown to orangish-brown sediments that appear to continuation of Context 4 into the new excavation area. Beginning of Urewe pottery detected at this horizon -but may be partially mixed outside of features within this layer.

Context 114: A poorly defined patch (25cm wide) of noticeably ashier sediment within the south side of the excavation, intersecting the unit wall.

Context 115: Rock lined circular feature with grey-brown softer ashier sediments within roughly 30cm north of 114 and clearly distinct. Feature labeled 115 and flotation given 116 number- but both contexts refer to the same feature.

Context 116: Small possible pit feature with slightly darker brown softer sediments within the plane of Context 103 in the eastern extension of the trench added to fully excavate feature 112.

Context 117: An elongate patch of darkened charcoal rich sediment that partially overlays the carbonate surface and extends from the southern edge of the current unit (2018 excavations) toward the Context 112 feature. While this feature surrounds Context 118/9 it is was not detected in 2018 and is not clearly visible in the 2018 profile even in 2020.

Context 118(9): Within 117 is the remaining half of a clear lens-shaped hearth feature bisected in 2018 (as Context 9) and still visible from the 2018 profile. Contexts 118 and 9 therefore refer to the northern and southern half (respectively) of the same hearth.

Context 119: An ashy darkened charcoal rich semi-circular feature (intersects wall) that sits on top of 112 feature with one sherd of likely Urewe incised ceramic within it.

Context 120: The extent of a burial pit within the plane of Context 103.1 in the eastern extension added to fully expose feature 112 (Burial 3- see Main Text for full details).

Context 121-121.2: Softer mottled grey-brown sediments with Kansyore type pottery that extend below contexts 120-123 in the southeastern corner of the trench against the back of the rock-wall (south and east of Context 112 feature).

Context 122: This is very compacted lighter brown sediment immediately below Context 112

Context 123: A pale circular (40cm across) lens of grey ashy sediment in the center of the unit within Context 122.

Context 124: Orangish-brown compacted sediment with mottled charcoal throughout underlying context 121. This was determined to be a cut partially overlaying 125.

Context 125-125.3: Similar to 124 matrix but grading into more frequent charcoal inclusions appearing first in the northwest end of the unit at the plane of 124, but extending below 124 across the entire plane of excavation and with much larger fragments of rock spall (60-100cm). Flotations from this plane produced a large quantity of finger millet grains, however these were all dated to the Iron Age and so represent intrusions. Larger charcoal fragments from this plane produced dates after 6000 cal. BP. The 125 matrix extends over 30cm.

Context 126: Southern portion of the unit with denser charcoal and more animal bone in a more compacted sediment, but otherwise identical to 125. Boundaries of 126 were diffuse and so not clear it represents a “feature”. Almost all of this area was taken for flotation.

Context 127: A dark brown ovoid feature within 125, oriented roughly east-west and beginning 60cm south of Context 126.

Context 128: This is the bottom few cm of Context 127 but distinct pale-grey color and softer ashier texture.

Context 129: Within 125.3, this is the area between two very large boulders in the southeast corner of the unit.

Context 130: Area on the plane of 125.3 but on the east (rockface) side of a large boulder in the northeast part of the unit.

Context 131: Underlying 125.3 matrix is a transition across the unit with increasing pale grey sediment, more granitic inclusions of all sizes, and substantial decrease in material culture. Multiple examples of partially articulated snake vertebrae and rodent remains occur across the trench.

Context 132: Context number not used.

Context 133-133.1: Continuation below 131 of paler grey/brown matrix with carbonate nodules and more compacted than 131. Artifact density continues to decrease. Large rockfall rubble now covers much of the unit and excavation stopped at this level everywhere except for the area of Context 130/134.

Context 134-134.3: Continuation below Context 130 of sediments in the northeastern corner of the unit where sediments continue down in a pocket between boulder rockfall. No artifacts are present but the area contains very dense mammal bone fragments until an arbitrary end of excavations at c. 210 cm below surface.

Context numbers 135-149, 151-153 are not used.

Context 150: Loose sediments associated with contexts 155/156 near the back of the shelter.

Context 155: Upper portion of the pocket of sediment behind the large partially-detached rockspall in the eastern edge of the unit (see Figure 6 -feature 3). Evidence for very significant insect bioturbation but rich in charcoal.

Context 156: Lower portion of the pocket of sediment behind large partially-detached rockspall (below 155) with paler sediment and very high density of large bovid long bones, bovid mandible, and several fragments of a large open-mouthed and spouted vessel.

**Trench IV**

Context 1: The top 20cm wass highly disturbed, consisting of fauna and Roulette ceramics. Context 1 was highly compacted from trampling by livestock. At an interface with a looser and darker horizon excavation transitioned to Context 1.1, as the material continued to be Roulette period ceramics with very few lithics. Both Context 1 and subdivision 1.1 had heavy rooting and some minor evidence of burrowing.

Context 2: This was a light brown heavily compacted horizon with mixed historic and prehistoric material. It was excavated in 10cm spits (Context 2, 2.1, and 2.2).

Context 2.2 Iron Age material increasingly abundant and the sediment transitioned to a more orangish brown color.

Context 3: This context began as Context 2.2 sediments began to transition to looser orange-brown sediments with a higher rate of granitic inclusions. Lithics were abundant, but there was very little pottery present. Large rocks increasingly common across the trench. Contains Context 4 (below). Subdivision Context 3.1 (below Contexts 3 and 4) was identical sediment, but featured a very dense accumulation of lithic artifacts and pottery in the northeast corner of Y5. Visual inspection of the plotted artifacts suggested this may be a “drop” zone, surrounded by a ring of “toss zone” lithic debris. In the center of this accumulation were several basalt cobbles with pitting suggestive of use as bipolar anvils and stones that may have been used as hammerstones. This area may have been an earlier occupational surface. It is likely that this was a lithic production area; it is not clear how it relates temporally to the evidence of a structure in Context 4.

Context 4: This was a feature within Context 3 composed of a darkened patch of sediment with an accumulation of large granite cobbles and several fragments of burnt and unburnt daga (clay construction material). Some of the daga fragments have pole impressions, indicating this material likely derives from a prehistoric structure, though no clear form or “wall line” was visible. Context 4 was also rich in charcoal and a large sample was taken for flotation. This was likely a secondary or otherwise post-depositional mixing deposit, as there was not a clear hearth feature.

Context 5: Very compacted brown sediment with abundant lithics, ceramics, and faunal remains. Faunal material occurred in concentrations in the east-middle edges of the trench. The bone in the unit had undergone diagenesis and was very fragile, fragmenting upon excavation. Given the compact and difficult to excavate nature of the sediment, it is likely that the water table rests (or rested) at this horizon for a long period of time. Excavation continued in arbitrary 10cm spits that continued to yield dense material culture and fauna. Context 5.2 encountered the top of a large animal burrow (Context 6), and began to reveal a large boulder in the eastern portion of the unit. Toward the end of Context 5 (in spits 5.3 and 5.4), it graded into increasingly orange and coarse sediments. Artifacts became less common and granitic slabs 10-40cm in length increased noticeably in frequency. It is likely that Context 5.4 should be considered the beginning of Context 7 based on this transition, but this was only noticed in the profile later. Bone preservation began to improve toward Context 7, again suggesting a true sediment transition. Ceramics were of a thicker black undecorated variant that did not resemble Kansyore, however later radiocarbon dates for this context indicate dates of c. 5000 BP.

Context 6: This was a very large burrow or pit feature (~30 cm wide, 50cm deep). It contained little material culture but many rodent bones, suggesting the presence of an animal burrow. Supporting this, the context was noticed as a cavity in Context 5 that “opened up” as it was excavated.

Context 7: This was a large ashy feature in Y5, it did not have a clear shape but all of the sediment was taken for flotation (Flot 35).

Context 8: Below Context 5 was very orange sandy silt that was looser overall than overlying sediment, but several harder patches were noted – especially in the southeastern corner. These patches seem to relate to areas of rockfall. Flakes in this context were larger and more commonly made on cherts and chalcedonies rather than quartz. The context contained no pottery and only a few bones, which were heavily stained orange. Several boulders were emerging at this point, limiting the area that could be excavated. A small patch of charcoal was taken for flotation, but yielded very little material. We ended excavations at an interface with sandier sediment similar to the eroding bedrock in Trench II.

**Trench V**

Context 1: This was the first 10-15cm of heavily compacted and very disturbed surface material, similar to Trench IV surface. It contained mostly fauna and historic Roulette period pottery. It also contained a large burnt feature labeled Context 2.

Context 2: This was a historic heath feature surrounded by a large patch of darkened burnt sediment with abundant large bovid bone and pottery. This likely reflects a historic meat feasting episode. The hearth was intersected by a very thick root, and in general Context 1 and 2 were subjected to heavy rooting throughout.

Context 3: Historic period materials continued, but likely Iron Age ceramics and more frequent lithics began to be encountered. This is likely a mixed and disturbed context, again similar to what was encountered in Trench IV. This context was excavated in 10cm spits. Context 3.3 contained a conical obsidian core and a fragment of iron slag. Excavations from this point continued only in the more northern FF13 square as excavation was becoming increasingly difficult. Context 3.4 contained a large grindstone on its side along the western edge of Y5, near the northwestern corner. A large flotation sample was taken from around the grindstone. Context 3.5-3.6 saw lithics continuing to increase but with no clear sediment change. Bone in this Context appears to have undergone the same form of diagenesis as bone in Context 5 of Trench IV.

Context 4: This context began with a very minor change in the matrix to a more orange and slightly less compacted sediment with larger and more frequent tabular granite inclusions. Artifact density decreased compared to previous contexts, and very little bone or pottery was present.

Context 5: This context saw the sediment became lighter and mostly sterile, with increasing proportions of tabular granite inclusions, Excavation was concluded after Context 5 due to lack of archaeological material as well as time constraints.

**Excavation profile descriptions**

**Trench II (North Profile)**

AAA: Fine silt with clay and minor sand inclusions, few pebbles. This a modern A horizon with extensive rooting. Few artifacts, all historic pottery. 10YR4/2.

BAA: Finer silt with no sand, but minor clay component. May be a later reworked occupational horizon creating a loose and more clay and organic rich level. No major inclusions except rare pebbles and granules, minor rooting. 10YR5/1.

BBA: Moderately compact, slightly darker grey sediment, more mottled with increase in pebbles and granules (differences in granules between layers likely related to rates of erosion/deposition of the granitic overhangs). This horizon captures major rock falls and has more extensive rooting. Horizon is level with Burial 2. 10YR4/1.

BBB: Lighter ashy silt with no sand in a small pit feature directly associated with Burial 2. Disturbed by major rooting that continued through the pelvis of Burial 2. 7.5YR5/1.

BCA: Compacted coarser silt with minor clay and sand. Granules and pebbles are common, cobbles are rare. Minor rooting. Likely a B horizon underlying Later Iron Age surface. 7.5YR4/4.

CAA: Very compacted sandy silt with very abundant granules, common pebbles and few cobbles. This is the top of eroding bedrock (C Horizon). Y.5YR3/3.

CAB: Diffuse and mottled boundary with CAA, but lighter sediment. Nearly solid rock at this point, crumbly granitic granules with some silt worked in. No large rocks within this layer.

**Trench III (South Profile***)*

See illustration in Figure 6

AAA: Compacted sandy silt surface. Trampled A horizon. Several burrows and other disturbances are visible. This excavation unit is in a more protected area further “back” in the rockshelter and much more rodent activity is visible here. 10YR3/2.

BAA: Fine silts with some clay component. Several large burrows are visible and layer appears to be mixed. Material culture is primarily historic and Later Iron Age pottery. 10YR3/3.

BAB, BAC, BAD: Upper, middle, and lower portion of a pit or burrow feature visible in the profile, but only visible as lightly grey coloration during excavations. All sediment is loose silts, and these divisions may reflect gradual filling in of the pit over time, suggesting it is likely a large natural burrow. Few artifacts were present in the small part of this feature which was in the excavated area.

BBA: Moderately compacted darker brown silt with common pebbles and granules. Resembles the B horizons elsewhere on the site, but in this trench this horizon is only evident in units N14 and N15. Artifacts are slightly mixed and include historic and prehistoric pottery. Animal bone and obsidian become more frequent.

UAA: Equivalent to BBA in stratigraphy but clearly separate. UAA is very anthropically derived, darker and more organic rich. Artifacts are more common and it includes dense overlapping hardened “floor: features- some broken up and reworked in the sediment. These may be from successive habitations set against the back of the rockshelter.

BCA: Underlying BBA in units N14 and N15 further from the rockshelter. This coarser more compacted silt with some clay and sand components. Granules and pebbles more common.

UBA: Under UAA, grading into lighter and more compacted sediments, very common Kansyore style pottery and lithics. Abundant bone especially toward the back of the shelter. Feature UBB is a dense accumulation of ash and refuse below UBA in the very back of the rockshelter.

UCA: Beneath UBA, Sediment is overall similar to UBA but granules, pebbles, common, larger fragments of rockfall increasingly common by this horizon. This level marks a clear transition to earlier deposits, with Kansyore pottery becoming abundant coincident with a dramatic increase in stone tool density. UCA contains several ash features including a clay-lined hearth and secondary ash dump feature.

UDA: Hardened surface that appears to be a “floor” that directly overlays the Kansyore Burial in this unit.

BDA: Semi-compacted orange sandy silt. Largely sterile with some rodent/reptile bones. Intensive rockfall (very abundant cobbles and boulders) prevented further excavation. Early Kansyore material present so may be Early Holocene. 7.5YR4/6.

**Trench IV (South Profile)**

See Figure 10

AAA: Very compacted slightly sandy silt with some clay component. This is the modern surface A horizon. 10YR3/2.

ABA: Compacted silt with clay, granite cobbles and pebbles are common, granules are very common. Dark organic rich horizon with mixed historic pottery. Heavily rooted. 10YR2/2. This is a slightly leached part of the A horizon.

BAA: Very compacted B horizon with little material culture. Primarily silt with rare pebbles and cobbles. Daga fragments are common. Grades gradually into BBA which is even more compacted and has a more pale color. Very little material culture aside from reworked daga. 10YR3/3.

BBA: Same sediment as BAA, but paler with more common material culture. 10YR4/3.

UAA: Little change from previous horizon in sediment- still silt but granules are more common with few pebbles. Several large cobbles are present in a possible feature related to a large spread of daga. High density of bone that has undergone diagenesis and is poorly preserved- likely due to fluctuations in the water table. Dense material culture and Urewe pottery. 10YR4/4.

BCA: Heavily compacted sandy silt. Grades into organish mottled sediments near the base. Granite cobbles, pebbles and granules all very common. Mostly lithic artifacts in this horizon. Some bone is well preserved under fragments of rockfall which are becoming more common. 7.5YR4/6.

CAA. Slightly sandly silt, looser and more sterile sediment similar to that overlaying bedrock in Trench 2. Very orange. Large slabs of rockfall prevent further excavation. 7.5YR4/6.

**Trench V (East Profile)**

AAA: Slightly sandy silt with a minor clay component. Loose to soft compaction. Few pebbles and granules. Relates to a modern A horizon. 10YR4/2.

ABA: Historic hearth feature. Very loose sandy silt that grades from pink to red surrounded by darker burnt sediments. Large roots visible in profile. 5YR6/4.

ABB: Slightly compacted ash lens underlying and related to ABA. 7.5YR5/1.

ABC: Disturbed mixed ABB/ABA/AAA material, likely from an animal burrow.

BAA/BAB: Heavily compacted B horizons with a very diffuse boundary. Color differences are only evident between the top (10YR4/3) and bottom (10YR3/3). Overall darker silt with more clay content- which seems to increase with depth. The deposition of clay is likely related to the resting water table. More charcoal is evident in the matrix within BAA, but granite granules and pebbles are very common throughout this B horizon.
