# Supplementary figures and images for: Cultural, economic, and settlement shifts over the last 9,000 years at Kakapel Rockshelter, Western Kenya
Source: PLoS One. 2025 Aug 20;20(8):e0328805. doi: 10.1371/journal.pone.0328805 (PMC12367187; doi:10.1371/journal.pone.0328805)

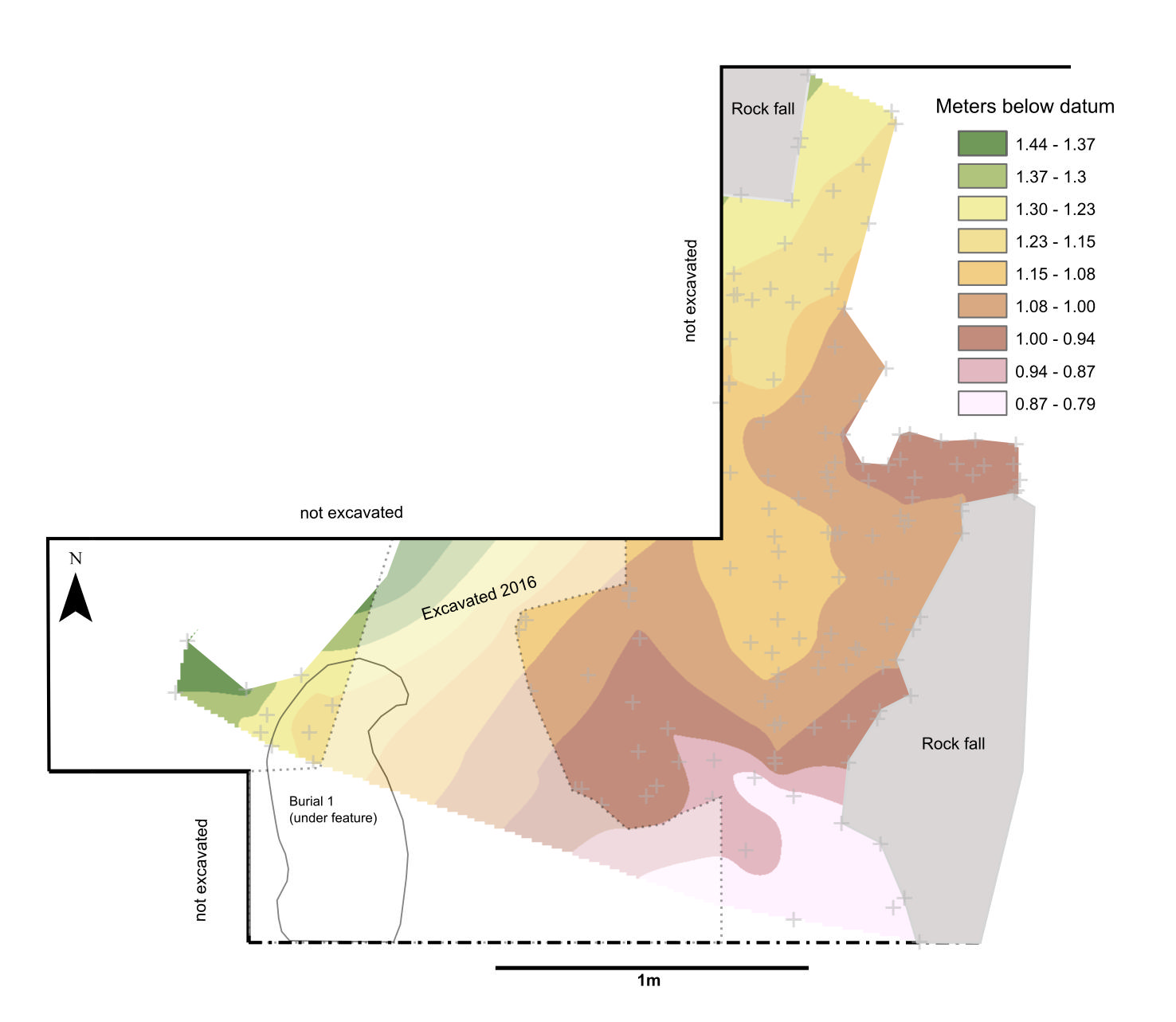

Supplement: S1 Fig — (TIF) [file pone.0328805.s002.tif]

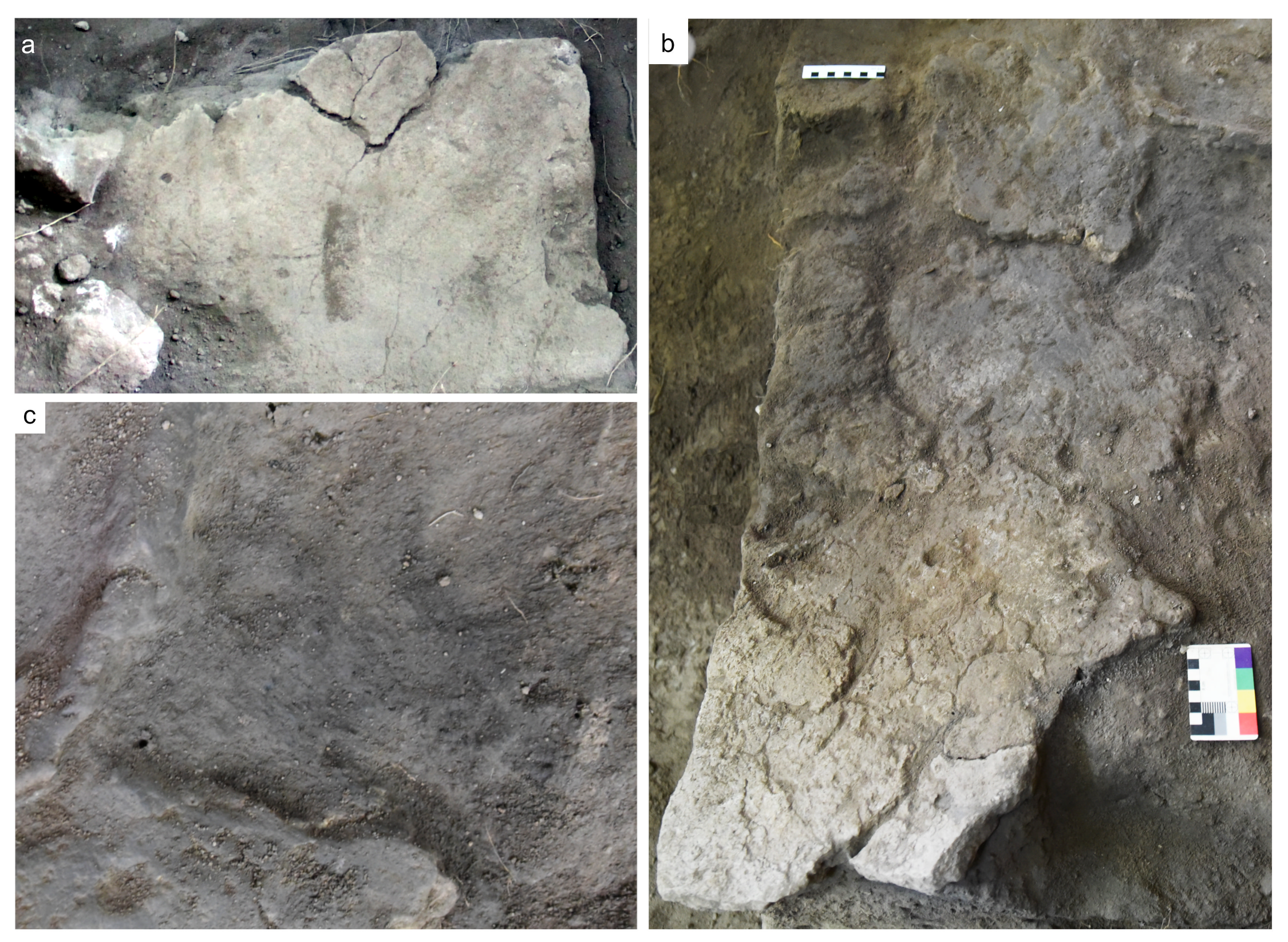

Supplement: S2 Fig — (TIF) [file pone.0328805.s003.tif]

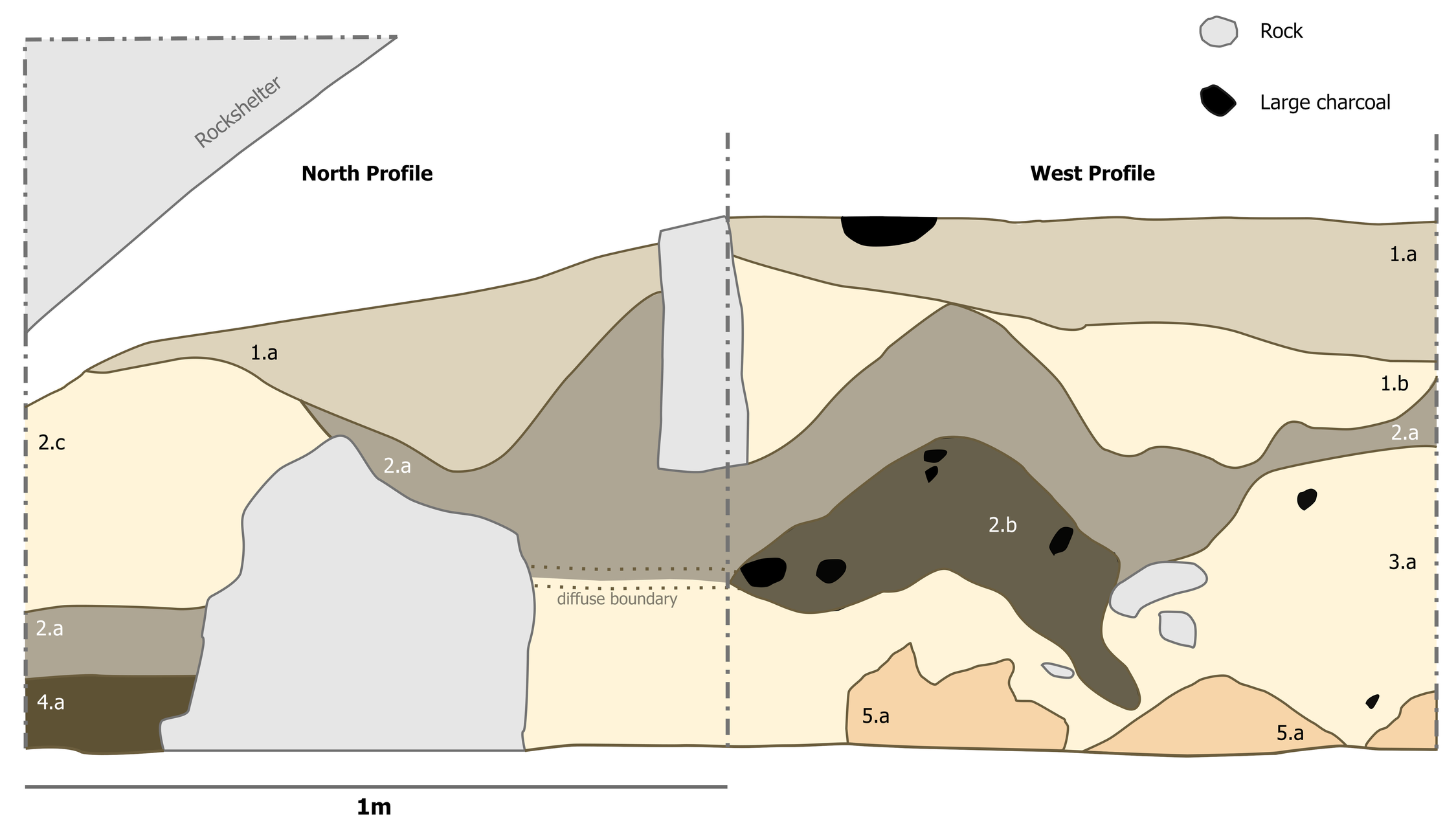

Supplement: S3 Fig — (TIF) [file pone.0328805.s004.tif]
